# Alternative splicing dynamics during human cardiac development *in vivo* and *in vitro*

**DOI:** 10.1016/j.stemcr.2025.102757

**Published:** 2026-01-02

**Authors:** Beatriz Gomes-Silva, Marta Furtado, Marta Ribeiro, Sandra Martins, Teresa Carvalho, André Ventura-Gomes, Henrike Maatz, Pragati Parakkat, Claudia Crocini, Michael Gotthardt, Rosina Savisaar, Maria Carmo-Fonseca

**Affiliations:** 1 Gulbenkian Institute for Molecular Medicine, Avenida Professor Egas Moniz, 1649-028 Lisboa, Portugal; 2 Faculdade de Medicina da Universidade de Lisboa, Avenida Professor Egas Moniz, 1649-028 Lisboa, Portugal; 3 Institute for Bioengineering and Biosciences and Department of Bioengineering, Instituto Superior Técnico, Universidade de Lisboa, Avenida Rovisco Pais, 1049-001 Lisboa, Portugal; 4 Max Delbrück Center for Molecular Medicine in the Helmholtz Association, Robert-Rössle-Strasse 10, 13125 Berlin, Germany; 5 Department of Cardiology, Charité-Universitätsmedizin Berlin, Hessische Strasse 3-4, 10115 Berlin, Germany; 6 DZHK (German Centre for Cardiovascular Research) Partner Site, Berlin, Germany; 7 Max Rubner Center for Cardiovascular Metabolic Renal Research, Deutsches Herzzentrum der Charité, Charité University Medicine Berlin, 10115 Berlin, Germany

**Keywords:** alternative splicing, pluripotent stem cells, cardiac myocytes, heart development

## Abstract

Cardiomyocytes differentiated *in vitro* from human induced pluripotent stem cells (iPSC-CMs) are increasingly used in studies of disease mechanisms, drug development, toxicity testing, and regenerative medicine. Alternative splicing (AS) plays a pivotal role in cardiac development. However, the extent to which iPSC-CMs recapitulate native cardiac splicing patterns remains poorly understood. Here, we provide a comprehensive temporal map of AS regulation during human cardiac development. iPSC-derived cardiomyocytes globally recapitulate the transcriptome of prenatal cardiomyocytes, yet their splicing profiles remain heterogeneous, with certain events reflecting early embryonic patterns and others resembling those of later-stage fetal hearts. Moreover, we uncover altered splicing events in iPSC-CMs, including mis-splicing of splicing factors. In conclusion, we present a resource of AS dynamics throughout human cardiac development and a catalog of splicing markers to assess cardiomyocyte maturation *in vitro*. Our findings provide critical insights into the limitations of iPSC-CM models and their utility in cardiovascular research.

## Highlights

• Define splicing transitions across human heart developmental stages
• Identify heterogeneous embryonic and fetal-like splicing patterns in iPSC-CMs
• Reveal aberrant splicing in iPSC-CMs impacting RNA-processing pathways
• Create a splicing reference to evaluate cardiomyocyte maturation *in vitro*

## Teaser

Carmo-Fonseca and colleagues chart splicing transitions across human heart development and compare them with splicing programs in iPSC-derived cardiomyocytes. They uncover mis-regulated splicing patterns in iPSC-CMs, particularly in RNA-processing pathways, and provide a benchmark resource to evaluate cardiomyocyte maturation *in vitro*.

## Introduction

Understanding human cardiac development is critical for advancing cardiovascular medicine and uncovering mechanisms underlying congenital and acquired heart diseases. Heart formation relies on the precise temporal control of gene expression programs, involving an evolutionarily conserved network of signaling pathways, transcription factors, and epigenetic alterations (Buijtendijk et al., 2020; Luna-Zurita et al., 2016; Olson, 2006; Sylva et al., 2014). These regulatory mechanisms govern the specification of cardiac cell fates and the differentiation and diversification of cardiac cell types throughout development (Meilhac and Buckingham, 2018).

A crucial mechanism that regulates gene expression during development is alternative splicing (AS), a process that expands transcript and protein diversity by generating multiple mRNA isoforms from a single gene (Nilsen and Graveley, 2010; Wang et al., 2008). Through AS of nascent transcripts (pre-mRNAs), individual genes produce a variety of mRNA species that may differ in stability, localization, or protein coding capacity (Braunschweig et al., 2014; Kjer-Hansen and Weatheritt, 2023; Yang et al., 2016). Alternatively spliced protein isoforms may have related, distinct, or even opposing functions (Kjer-Hansen and Weatheritt, 2023; Yang et al., 2016). Tissue-specific regulation of AS plays a crucial role in defining the identity and function of adult tissues, and its regulation is finely tuned during development (Baralle and Giudice, 2017; Blencowe, 2006; Merkin et al., 2012).

AS plays a pivotal role in cardiac cell differentiation and maturation, with numerous splicing isoforms emerging at different developmental stages (Giudice et al., 2014; Kalsotra et al., 2008; Van Den Hoogenhof et al., 2016). Recently, a cross-species comparison of splicing patterns across pre- and postnatal development of multiple organs revealed that splicing regulation is fundamental for heart development (Mazin et al., 2021). Despite these insights, a comprehensive characterization of AS during human cardiac development remains lacking. Furthermore, the physiological roles of developmentally regulated splicing isoforms are still poorly understood, limiting our ability to fully appreciate how splicing transitions contribute to heart formation and function.

Major advances in cardiac developmental biology have resulted from studies in murine models (Burridge et al., 2015). However, despite their utility, rodent heart architecture and cell function are very different from humans (Zaragoza et al., 2011). The discovery that differentiated adult human somatic cells can be reprogrammed into a pluripotent state (Takahashi et al., 2007) revolutionized the field, enabling the directed differentiation of pluripotent stem cells into well-defined cardiac lineages *in vitro*. Currently, human induced pluripotent stem cell-derived cardiomyocytes (iPSC-CMs) are widely used as an alternative to animal models for disease modeling, drug discovery, and toxicity screening (Karbassi et al., 2020). Moreover, iPSC-CM transplantation is being explored as a potential strategy to repopulate damaged myocardial tissue and restore heart function (Wang et al., 2023).

Although iPSC-CMs are generally considered to resemble fetal cardiomyocytes (Karbassi et al., 2020), a systematic global comparison of splicing programs between iPSC-CMs and human hearts has yet to be performed. Such an analysis is crucial to understanding the extent to which iPSC-CMs recapitulate native cardiac splicing transitions and to identifying potential limitations in their maturation.

In this study, we conducted a comparative analysis of AS patterns in iPSC-CMs and human hearts across developmental stages, from early organogenesis to adulthood. Our findings provide a resource of developmentally regulated AS events, capturing splicing transitions that occur as the heart progresses from embryonic to postnatal life. While splicing profiles in iPSC-CMs globally resemble those of prenatal heart samples, we uncovered a subset of splicing events unique to iPSC-CMs, including mis-splicing of genes involved in RNA processing. Together, these findings provide a comprehensive characterization of AS dynamics during human heart development and establish a catalog of splicing events that can serve as benchmarks for assessing cardiomyocyte maturation *in vitro*.

## Results

### Genome-wide comparison of splicing profiles in iPSC-CMs and developing hearts

**Figure 1:**
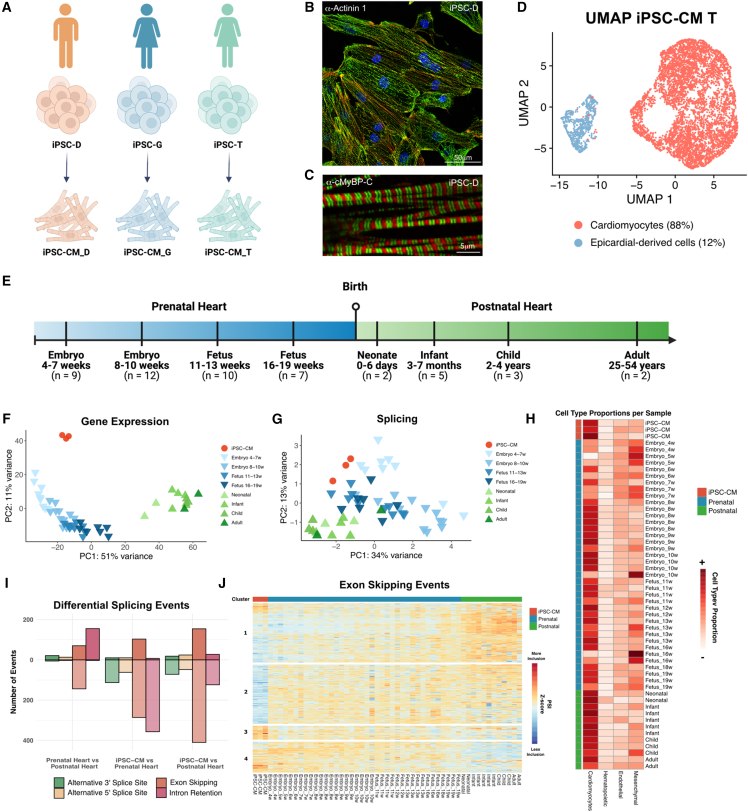
Genome-wide comparison of splicing profiles in iPSC-CMs and developing hearts (A) Schematic representation of iPSC differentiation into iPSC-CMs. The iPSC cell lines and their respective sexes are indicated. (B and C) Immunofluorescence images showing the expression of (B) α-Actinin 1 and (C) α-MyBP-C, in iPSC-CMs on day 30 of differentiation. Samples were derived from the indicated iPSC lines. Nuclei are stained with DAPI. Scale bar included in each image. (D) UMAP plot of iPSC-CMs derived from iPSC-T, revealing two major subpopulations of cells corresponding to cardiomyocytes (red) and epicardial-derived cells (blue). (E) Schematic representation of the developing human heart RNA-seq dataset, with the corresponding time points. (F and G) Principal-component analysis based on the (F) 500 most variable protein-coding genes and (G) 500 most variable splicing events, across iPSC-CMs (in red), prenatal hearts (in blue), and postnatal hearts (in green). Lighter shades represent younger samples, and darker shades correspond to older samples. Splicing events in (G) include exon skipping, intron retention, and alternative 3′ and 5′ splice sites detected by vast-tools. (H) Heatmap showing estimated cell-type proportions in heart and iPSC-CM samples, determined using CIBERSORTx. (I) Barplot depicting the total number of differentially spliced events (ΔPSI ≥ ±0.2, adjusted *p* value ≤0.01) in comparisons between Prenatal vs. Postnatal Heart, iPSC-CM vs. Prenatal Heart, and iPSC-CM vs. Postnatal Heart. Events above 0 (darker color) indicate higher inclusion in the first group of each comparison, while events below 0 (lighter color) indicate lower inclusion in the first group. (J) Heatmap displaying *Z* score normalized percent spliced in (PSI) levels for all exon skipping events, comparing iPSC-CMs with prenatal hearts, iPSC-CMs with postnatal hearts, and prenatal hearts with postnatal hearts. Colors reflect inclusion levels relative to the mean of each event (red for higher inclusion, blue for lower inclusion). Event-specific information is provided in Tables S2 and S3.

To evaluate whether iPSC-CMs recapitulate the splicing patterns of the heart and determine the developmental stage they most closely resemble, we directed the differentiation of three independent iPSC lines (referred to as D, G, and T; Figure 1A) into cardiomyocytes (see Methods). The majority of differentiated iPSC-CMs exhibited cardiomyocyte-like morphology, including an elongated shape and well-defined sarcomere structures (Figures 1B and 1C).

To assess which cell types were present in monolayer cultures of iPSC-CMs at day 30 of differentiation, we conducted single-cell RNA sequencing (sequencing metrics in Table S1). The results revealed that 84%–88% of cells expressed gene signatures characteristic of cardiomyocytes (Figures 1D and S1A), including the expression of sarcomeric and mitochondrial genes (Figures S1B and S1C). In contrast, ∼12%–16% of cells were enriched in *TNNT1*, the predominant troponin isoform during early cardiac development, as opposed to *TNNT2*, which is characteristic of fully mature cardiomyocytes (Figure S1B). Label transfer annotation using the Farah et al. fetal heart single-cell atlas (Farah et al., 2024) confirmed these cells resembled the gene expression profile of epicardial cells (Figure S1D). Accordingly, these cells displayed expression of epicardial markers, including *WT1*, *UPK3B*, and *ALDH1A2* (Figure S1B) (Cao et al., 2020; Cui et al., 2019). The epicardial-like cells were enriched in extracellular-matrix-related pathways, whereas the cardiomyocyte population showed enrichment in cardiac development pathways (Figure S1E). This observation aligns with studies reporting the bifurcation of cardiac progenitors into myocardial and epicardial lineages during both iPSC differentiation (Galdos et al., 2023) and mouse cardiac development (Tyser et al., 2021).

Next, we conducted bulk RNA-seq of polyadenylated RNA isolated from the three iPSC-CM lines at day 30 of differentiation (sequencing metrics in Table S1). To assess potential variability among iPSC-CMs derived from unrelated individuals, we performed pairwise comparisons of gene expression profiles across the three lines (Figure S1F). This analysis revealed a high degree of similarity across the lines, with correlation coefficients comparable or even exceeding those observed between two embryonic heart samples at the same developmental stage (Figure S1F), suggesting that the differentiation process yields highly similar iPSC-CMs regardless of donor origin. Additionally, we observed a substantial overlap in expressed genes among all three lines (Figure S1G), reinforcing the consistency of the transcriptional landscape across replicates.

To understand how the transcriptomes of iPSC-CMs relate to gene expression profiles during heart development, the three datasets from iPSC-CM cultures were grouped as biological replicates and compared with publicly available bulk RNA-seq datasets from human heart samples, spanning from early organogenesis to adulthood (Cardoso-Moreira et al., 2019). This dataset comprises 38 prenatal samples collected between 4- and 19-week post-conception and 12 postnatal samples collected from newborns, infants, young children, and adults (Figure 1E). To explore the primary sources of variation in gene expression profiles of iPSC-CMs and human hearts at distinct developmental stages, we performed principal-component analysis (PCA). This analysis revealed that the first principal component (PC1), accounting for 51% of the variance, effectively distinguishes prenatal heart samples from postnatal samples, capturing progressive transcriptional changes throughout development (Figure 1F). Along PC1, the iPSC-CMs clustered most closely with 8- to 10-week prenatal heart samples (Figure 1F). Hierarchical clustering revealed that the iPSC-CMs cluster closer to the prenatal heart than to the postnatal heart samples, and bootstrapping confirmed the reliability of the clustering, attributing 100% confidence (approximately unbiased *p* value × 100) to the node that clusters the iPSC-CMs with the prenatal hearts (Figure S1H).

To further assess the developmental state of iPSC-CMs, we leveraged our RNA-seq data to examine the expression of genes associated with key metabolic pathways involved in cardiomyocyte maturation. Metabolic maturation is characterized by shift from anaerobic glycolysis to mitochondrial oxidative phosphorylation (OXPHOS), accompanied by increased fatty acid β-oxidation (FAO) and mitochondrial biogenesis (Maroli and Braun, 2021). Using single-sample gene set enrichment analysis (ssGSEA), we confirmed that prenatal heart samples showed higher glycolytic activity and lower OXPHOS, while postnatal hearts showed the reverse pattern, consistent with metabolic remodeling (Figure S1I). iPSC-CMs retained a glycolytic, prenatal-like metabolic profile (Figure S1I). To further refine this analysis, we curated cardiomyocyte-relevant gene panels for glycolysis, the TCA cycle, OXPHOS, and FAO, and visualized their expression in a combined heatmap (Figure S1J). While iPSC-CMs express key components of the TCA cycle and OXPHOS machinery, similar to late-prenatal hearts, the expression of FAO-related genes remained markedly low, even compared to early prenatal hearts (Figure S1J). These findings align with prior studies (Jiang et al., 2024; Vučković et al., 2022) and suggest that although iPSC-CMs possess functional mitochondria capable of oxidative phosphorylation, they fail to activate the FAO program, a hallmark of metabolically mature cardiomyocytes.

To identify and quantify AS, we used rMATS, MAJIQ, and vast-tools, focusing on the most common patterns of AS events (i.e., exon skipping, alternative 3′ and 5′ splice sites, and intron retention). Pairwise comparisons of the inclusion levels of all AS events detected in the three iPSC-CM cultures revealed Spearman correlation coefficients of ∼0.93, higher than that observed between embryonic hearts at the same developmental age (Figure S1K). The three iPSC-CM datasets were grouped and compared with heart datasets. While PCA of splicing data was noisier than that of gene expression, considering that developmental time is captured by the combination of the two principal components, iPSC-CMs consistently clustered closer to the prenatal samples, across all three tools (Figures 1G, S1L, and S1M). Given the distinct cell-type composition of iPSC-CMs and heart tissues, differences in splicing patterns could be influenced by sample heterogeneity. To explore this possibility, we applied CIBERSORTx to estimate the relative abundance of major cardiac cell types in our bulk RNA-seq datasets using a single-cell reference. This analysis revealed that iPSC-CMs were highly enriched in cardiomyocytes, consistent with their purification by fluorescence-activated cell sorting with VCAM1 antibodies at day 13 of differentiation, whereas prenatal heart samples exhibited greater cellular diversity and a comparatively lower proportion of cardiomyocytes (Figure 1H). While CIBERSORTx does not provide definitive cell-type quantification, it offers a useful approximation to assess whether compositional differences might influence transcriptome-wide splicing patterns. As the cardiomyocyte content of iPSC-CMs was more similar to postnatal than to prenatal samples (Figure 1H), it is thus unlikely that their transcriptional clustering with prenatal hearts is solely driven by cell-type composition.

Pairwise differential splicing analysis, focusing on identifying events with a deltaPSI (difference in percent spliced in) higher than 20%, revealed 411 differentially spliced events between prenatal and postnatal heart samples (Figure 1I; Table S2). When comparing iPSC-CMs and heart samples, we found 948 splicing events differing between *in vitro* cultures and prenatal samples and 877 events dissimilar between *in vitro* differentiated cardiomyocytes and postnatal heart samples (Figure 1I; Table S3). Alternative ‘‘cassette’’ exon and intron retention were the most frequent types of alternative splicing identified (Figure 1I).

Hierarchical clustering of differentially spliced events revealed distinct patterns of exon usage and intron retention across developmental stages. Exons predominantly skipped in prenatal hearts were grouped in cluster 1, while those primarily skipped in postnatal hearts were grouped in cluster 4 (Figure 1J). Notably, within cluster 1, the AS events in iPSC-CMs segregated into two subgroups: a smaller upper subgroup with splicing patterns more closely resembling postnatal hearts and a larger lower subgroup aligning more closely with prenatal profiles, highlighting the heterogeneous nature of AS in iPSC-CMs. The analysis also identified exon skipping events that uniquely distinguish iPSC-CMs from both prenatal and postnatal hearts (Figure 1J, clusters 2 and 3), as well as intron retention events specific to iPSC-CMs (Figure S1N, cluster 2). Finally, a skew toward increased intron retention was observed in prenatal hearts (Figure S1N, cluster 3).

Taken together, our findings indicate that iPSC-CMs globally resemble prenatal cardiac cells at both the transcriptional and splicing levels, although some variability is observed across individual splicing events.

### Pre- to postnatal splicing transitions in the heart

Among the 411 events differentially spliced between prenatal and postnatal heart samples (Table S2), most had not been previously reported as developmentally regulated during human cardiac maturation *in vivo* and were predicted to have functional consequences at the protein level (Table S4). To infer the cell-type specificity of the genes affected by these splicing events, we analyzed their expression using published scRNA-seq data from human hearts (Farah et al., 2024). This analysis showed that many of the newly identified events occur in genes that are either predominantly expressed in non-cardiomyocyte populations or are broadly expressed across multiple cardiac cell types. Thus, to avoid potential misinterpretation due to differences in cellular composition, we excluded these events from comparisons with iPSC-CM samples.

**Figure 2:**
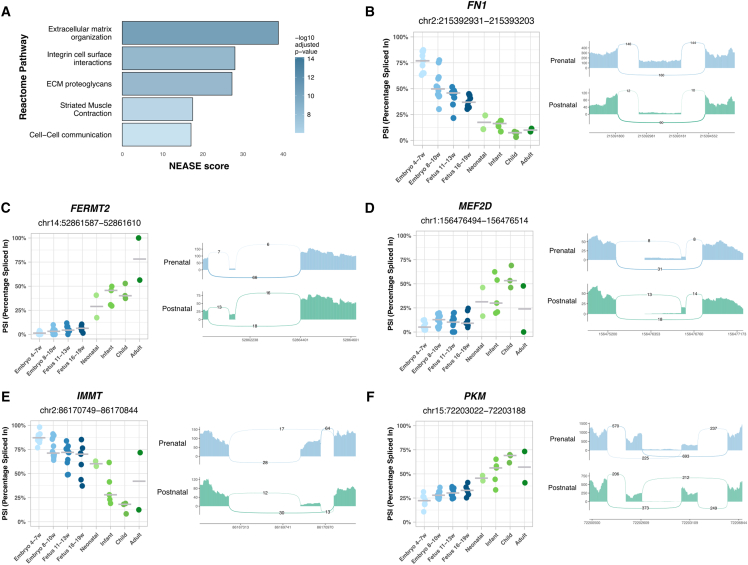
Pre- to postnatal splicing transitions in the heart (A) Top 5 enriched Reactome pathways identified by NEASE for differentially spliced exons identified between prenatal and postnatal hearts. (B–F) Inclusion levels (PSI) of exons differentially included between prenatal and postnatal hearts, specifically (B) *FN1* exon 25 (*FN1*-203), (C) *FERMT2* exon 11 (*FERMT2*-215), (D) *MEF2D* exon 8 (*MEF2D*-201), (E) *IMMT* exon 6 (*IMMT*-204), and (F) *PKM* exon 9 (*PKM*-219). PSI levels are shown for prenatal hearts and postnatal hearts. Each event is accompanied by the corresponding sashimi plot.

To investigate the potential functional significance of the newly identified events, we used *VastDB* and *NEASE* to predict their effects on protein function and interactions. A striking enrichment of splicing events predicted to impact proteins associated with the extracellular matrix (ECM) was observed (Figure 2A). Among affected ECM genes, we observed alternative splicing of fibronectin (*FN1*) exons 25 (Figure 2B) and 33 (Figure S2A), corresponding to the EDB and EDA isoforms (Efthymiou et al., 2021). We also observed AS changes in Kindlin-2 (*FERMT2*), a regulator of integrin-mediated cell adhesion and ECM remodeling (Godbout et al., 2020). In the postnatal heart, we found increased inclusion of exon 11 (Figure 2C), which encodes a domain involved in various protein interactions. Relative to pre-natal hearts, we also found increased inclusion of Fibulin-2 (*FBLN2*) exon 9 (Figure S2B), which encodes a calcium-binding EGF domain (Timpl et al., 2003).

We further detected splicing changes in the transcription factor *MEF2D*, with increased inclusion of exon 8, which encodes a domain essential for transcriptional activation (Gönczi et al., 2023), occurring after birth (Figure 2D). Mutually exclusive alternative splicing produces two major *TCF3* isoforms that differ only in their DNA-binding domain (Yamazaki et al., 2018). During heart development, the isoform E47-specific exon was more included after birth (Figure S2C). Increased inclusion of *MLF1* exon 3, which disrupts a domain required for cell-cycle control (Yoneda-Kato et al., 2005), was further detected in postnatal hearts (Figure S2D).

Additionally, we identified AS events in metabolism-related genes. We observed a progressive decrease in inclusion of *IMMT* exon 6 during development, particularly after birth (Figure 2E). *IMMT* encodes the mitochondrial protein mitofilin (John et al., 2005), and its exon 6 encodes a domain required for the function of mitochondrial cristae (Bock-Bierbaum et al., 2022). We also detected an AS switch in *PKM* (Figure 2F). This gene encodes pyruvate kinase M, an enzyme that catalyzes the final step in glycolysis. Exons 9 and 10 of *PKM* are mutually exclusive, giving rise to two isoforms with distinct metabolic roles: exon 9 inclusion produces PK-M1, an isoform predominantly expressed in terminally differentiated tissues with high oxidative demands, while exon 10 inclusion generates PK-M2, an isoform commonly found in proliferative fetal tissues and cancer (Christofk et al., 2008). Our finding that *PKM* exon 9 inclusion is higher in postnatal hearts compared to prenatal tissues (Figure 2F) suggests that this splicing transition contributes to the metabolic shift from glycolysis to oxidative phosphorylation during cardiac maturation after birth.

In summary, we identified numerous novel cardiac AS events that are regulated during the transition from pre- to postnatal life. These splicing alterations are predicted to affect critical cardiac functions, including extracellular matrix remodeling, transcriptional control, and metabolic adaptation.

### Splicing regulation in the developing prenatal heart

While the transition to postnatal life is a critical point for splicing regulation, splicing patterns during the prenatal period are not uniform across developmental stages. A comparison of early embryonic (4–5 weeks post-conception) and later fetal (18–19 weeks post-conception) heart samples revealed 563 alternative exon skipping events and 73 intron retention events (Table S5).

**Figure 3:**
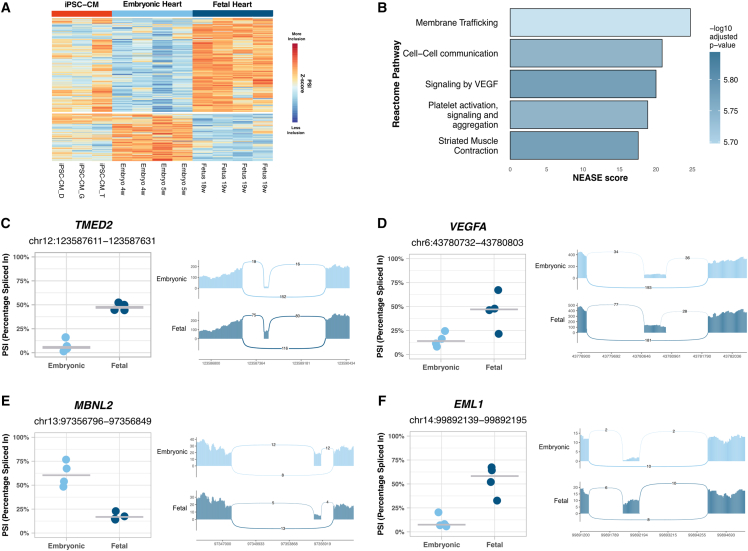
Splicing regulation in the developing prenatal heart (A) Heatmap displaying *Z* score normalized percent spliced in (PSI) levels of all exon skipping events identified when comparing 4–5 weeks embryonic with 18–19 weeks fetal hearts. Colors represent inclusion levels relative to the mean for each event (red indicates higher inclusion, blue indicates lower inclusion). Event-specific information is provided in Table S5. (B) Top 5 enriched Reactome pathways identified by NEASE for differentially spliced exons identified between 4 and 5 weeks embryonic and 18–19 weeks fetal hearts. (C–F) Inclusion levels (PSI) of exons differentially spliced between 4 and 5 weeks embryonic and 18–19 weeks fetal hearts, specifically (C) *TMED2* exon 3 (*TMED2*-202), (D) *VEGFA* exon 6 (*VEGFA*-226), (E) *MBNL2* exon 6 (*MBNL2*-207), and (F) *EML1* exon 2 (*EML1*-222). PSI levels are shown for embryonic hearts and fetal hearts. Each event is accompanied by the corresponding sashimi plot.

Embryonic hearts showed higher levels of exon skipping (Figure 3A) and intron retention (Figure S3A) compared to fetal hearts. Genes undergoing differential splicing during the embryonic to fetal transition were most strongly enriched in pathways associated with membrane trafficking, cell-cell communication, and vascular endothelial growth factor (VEGF) signaling (Figure 3B; Table S6).

For instance, *TMED2*, a gene involved in cargo selection and vesicle formation and essential for development (Di Minin et al., 2022), begins to include its heart-specific micro-exon 3 in the fetal stage (Figure 3C).

*VEGFA* exon 6, located in the heparin-binding domain, was more included in fetal compared to embryonic hearts (Figure 3D).

*MBNL2* exon 6, which encodes a nuclear localization signal, showed higher inclusion in embryonic hearts compared to fetal hearts (Figure 3E).

*EML1* exon 2, which encodes a motif thought to mediate interactions with microtubules and membranes, was more included in fetal hearts compared to embryonic hearts (Figure 3F).

Additional genes with significant regulation of prenatal splicing included *MACF1*, *LAMA2*, *ANK3*, *ABI1*, *DCAF6*, *SLC25A3*, and *ATP2B4* (Figures S3B–S3H; Table S6).

In conclusion, our findings demonstrate that cardiac splicing is dynamically regulated throughout prenatal development.

### Heterogeneous recapitulation of developmental splicing regulation in iPSC-CMs

**Figure 4:**
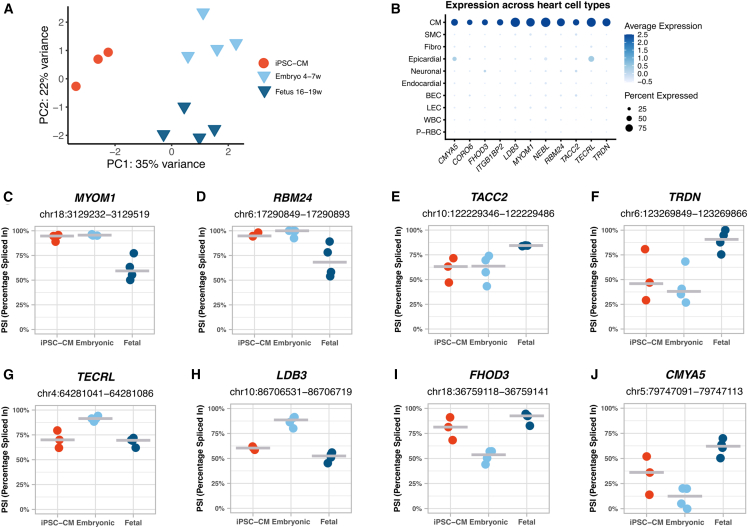
Heterogeneous mirroring of prenatal heart splicing regulation in iPSC-CMs (A) PCA of the 500 most variable splicing events in iPSC-CMs (red), 4–5 weeks embryonic hearts (in light blue), and 18–19 weeks fetal hearts (in dark blue), as identified by MAJIQ. (B) Dot plot depicting the expression of cardiomyocyte-specific genes (log2FC > 0.5 compared with all other cell types, expressed in >50% of cardiomyocytes and <10% of other cell types) across the fetal heart single-cell atlas. Dot size represents the percentage of cells expressing each gene, and dot color indicates the scaled expression levels averaged per cell population. (C–J) Inclusion levels (PSI) of exons differentially spliced between 4 and 5 weeks embryonic and 18–19 weeks fetal hearts, specifically (C) *MYOM1* exon 18 (*MYOM1*-202), (D) *RBM24* exon 4 (*RBM24*-207), (E) *TACC2* exon 15 (*TACC2*-210), (F) *TRDN* exon 31 (*TRDN*-201), (G) *TECRL* exon 11 (*TECRL*-201), (H) *LDB3* 8 (*LDB3*-202), (I) *FHOD3* exon 26 (*FHOD3*-206), and (J) *CMYA5* exon 5 (*CMYA5*-201). PSI levels are shown for iPSC-CMs, embryonic hearts, and fetal hearts.

Although PCA analysis segregated embryonic and fetal hearts along PC2, with iPSC-CMs clustering in between (Figure 4A), direct comparisons are confounded by the cellular heterogeneity of prenatal heart tissue, which contains a substantial proportion of non-cardiomyocytes. To mitigate this limitation, we restricted the analysis to genes predominantly expressed in cardiomyocytes. Based on single-cell transcriptomic data from the fetal heart atlas (Farah et al., 2024), we identified 491 cardiomyocyte-enriched genes (log2FC > 0.5 compared to all other cell types), of which 86 met stricter criteria for specificity (expression in >50% of cardiomyocytes and <10% of non-cardiomyocytes). Within these cardiomyocyte-enriched genes (Table S7; Figures 4B and S4A), we detected 79 AS events regulated during prenatal development. Comparison of these events revealed broad variability between iPSC-CMs and prenatal hearts. In some cases, iPSC-CM splicing patterns closely mirrored those of embryonic hearts (Figures 4C–4F and S4B–S4D), whereas other events were more similar to fetal hearts (Figures 4G and S4E–S4G) or displayed intermediate profiles (Figures 4H–4J). Strikingly, even within the same gene, different AS events could align with distinct developmental stages (Figures S4H–S4O). For a subset of AS events, the pattern in iPSC-CMs diverged from that of embryonic and fetal hearts (Figures S4P–S4Q), suggesting a more mature splicing profile *in vitro*. Together, these findings indicate that iPSC-CMs display a heterogeneous maturation state with respect to developmental splicing regulation in the prenatal heart.

### Maturation-driven splicing changes in iPSC-CMs recapitulate developmental transitions in the human heart

**Figure 5:**
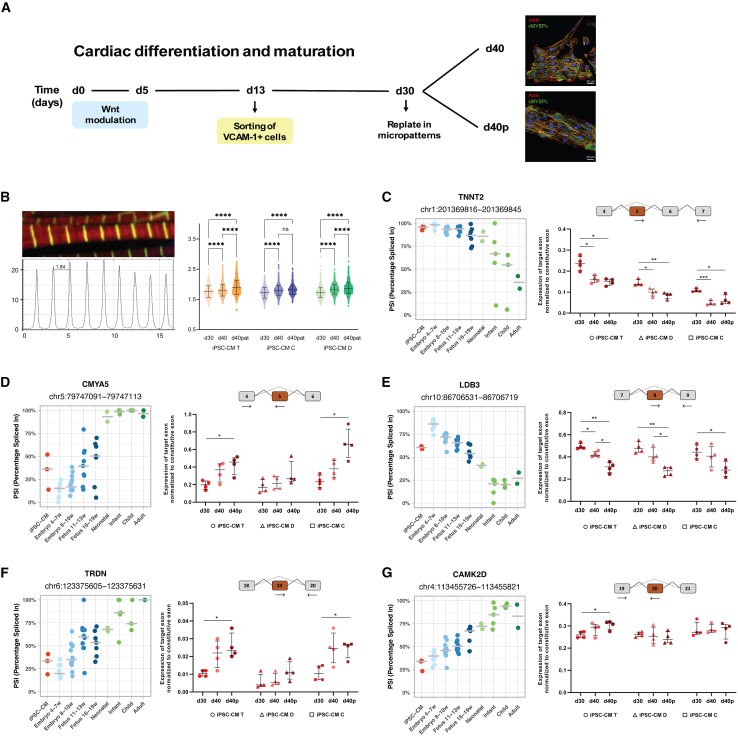
Maturation-driven splicing changes in iPSC-CMs recapitulate developmental transitions in the human heart (A) Diagram illustrating the differentiation protocol used to generate iPSC-CMs and the experimental conditions for assessing alternative splicing during *in vitro* maturation. Conditions include iPSC-CMs at 30 days of differentiation, 40 days of differentiation, and 40 days of differentiation with micropatterning. (B) Immunofluorescence image showing expression of α-Actinin 1 (green) and Actin (red) in sarcomeres of iPSC-CMs at day 30 of differentiation. Plot profile of α-Actinin 1 fluorescence intensity across sarcomeres. Sarcomere length measurements of iPSC-CMs derived from three cell lines (T, D, and C) across three experimental conditions. Statistical significance is denoted as ∗∗∗∗, indicating a *p* value < 0.0001. (C–G) Inclusion levels (PSI) of exons differentially spliced between prenatal and postnatal hearts, specifically (C) *TNNT2* exon 5 (*TNNT2*-224), (D) *CMYA5* exon 5 (*CMYA5*-201), (E) *LDB3* exon 8 (*LDB3*-202), (F) *TRDN* exon 19 (*TRDN*-201), and (G) *CAMK2D* exon 18 (*CAMK2D*-202). PSI levels are shown for iPSC-CMs, prenatal hearts, and postnatal hearts across various developmental time points. Each splicing event is accompanied by *in vitro* PCR quantification performed in three distinct cell lines (T, D, and C) under three experimental conditions: 30 days of differentiation (in red), 40 days of differentiation (in pink), and 40 days of differentiation with micropatterning (in dark red). Significance levels are indicated as ∗(*p* < 0.05), ∗∗(*p* < 0.01) and ∗∗∗(*p* < 0.001).

The observation that iPSC-CMs mimic prenatal heart splicing patterns but vary in their resemblance to specific developmental stages suggests heterogeneity in splicing trajectories during *in vitro* differentiation. To investigate this, we selected five splicing events for analysis by RT-qPCR at different stages of iPSC-CM maturation. We designed two experimental setups to assess how differentiation time and mechanical environment influence splicing regulation in iPSC-CMs. In one set of experiments, iPSC-CMs differentiated for 30 days were maintained in culture for an additional 10 days (Figure 5A). In another set of experiments, iPSC-CMs differentiated for 30 days were cultured for an additional 10 days on a micropatterned surface (Figure 5A). We analyzed cardiomyocytes derived from three independent iPSC lines, performing three separate differentiation experiments for each line. As a hallmark of maturation, we assessed sarcomere length (Figure 5B). We observed a significant increase in sarcomere length between day 30 and day 40 (Figure 5B). Except for iPSC-CM C, culture on a micropatterned surface further increased the sarcomere length (Figure 5B).

For *TNNT2* exon 5, which increases calcium sensitivity and is predominantly included in prenatal hearts (Gomes et al., 2002), we observed a reduction in inclusion levels at day 40 compared to day 30. Culturing iPSC-CMs on a micropatterned surface did not further influence exon 5 exclusion (Figure 5C).

In *CMYA5*, which encodes a protein required for cardiac dyad architecture (Lu et al., 2022), we identified novel exon skipping and intron retention events in prenatal hearts (Figures 5D and S5A) that are predicted to disrupt the reading frame and trigger nonsense-mediated decay. Consistent with this prediction, the levels of *CMYA5* mRNA were significantly reduced in prenatal hearts (Figure S5B). In hearts, inclusion of exon 5 increased progressively during prenatal development (Figure 5D). In iPSC-CMs, we observed a similar trend, with exon 5 inclusion increasing between day 30 and day 40 of differentiation. This effect was particularly pronounced in cells cultured on a micropatterned surface (Figure 5D).

We identified several exons in the sarcomeric Z-line protein gene *LDB3* whose inclusion is developmentally regulated in the heart (Figures 5E and S5C–S5E) (Huang et al., 2003). Inclusion of exon 8 decreased progressively during prenatal development (Figure 5E). A significantly reduced inclusion of this exon was also observed in iPSC-CMs between day 30 and day 40, and this effect was more pronounced in iPSC-CMs cultured on micropatterned surfaces (Figure 5E).

For *TRDN*, a key regulator of calcium release (Chopra and Knollmann, 2013), we identified a progressive increase in exon 19 inclusion during prenatal heart development (Figure 5F). A similar trend of increased inclusion was observed in iPSC-CMs between day 30 and day 40 (Figure 5F).

For *CAMK2D*, a gene involved in calcium handling and transcriptional regulation (Duran et al., 2021), exon 18 inclusion increased progressively during heart development (Figure 5G). Surprisingly, we detected little to no modulation of exon 18 inclusion in iPSC-CMs between day 30 and day 40, regardless of whether the cells were cultured on micropatterned surfaces (Figure 5G). These findings suggest that the regulation of exon 18 inclusion in *CAMK2D* during development is not replicated in iPSC-CMs.

In conclusion, our results demonstrate that, despite inherent variability across iPSC lines and differentiation experiments, both temporal progression in culture and mechanical cues from micropatterned surfaces significantly influence splicing regulation. Except for *CAMK2D*, we observed a consistent trend of recapitulating the developmental splicing transitions characteristic of *in vivo* heart development.

To evaluate whether developmentally regulated alternative transcripts are translated into distinct protein isoforms, we performed deep proteomic profiling of iPSC-CMs. Two of the iPSC lines used for proteomic analysis were the same as those profiled by RNA-seq (T and D); however, iPSC line G was replaced by line C due to sample availability. All iPSCs were differentiated to cardiomyocytes using the same protocol. In parallel, we analyzed previously published high-resolution proteomic datasets from healthy adult human hearts (Doll et al., 2017). Our strategy for detecting translated isoforms focused on AS events that are developmentally regulated and occur in genes predominantly expressed in cardiomyocytes (Table S8). As an illustrative example, AS of exon 18 in the *MYOM1* gene results in isoform-specific peptides: exon inclusion gives rise to peptides spanning exons 17–18 and 18–19, while exon skipping produces a junctional peptide spanning exons 17–19. In iPSC-CMs, we detected 4 MS/MS spectra supporting translation of isoforms resulting from exon inclusion. In adult heart samples, peptides corresponding to both isoforms were detected, with a predominance of exon-skipping peptides (Table S8). This is consistent with the RNA-seq observation that exon 18 is largely excluded from *MYOM1* transcripts in the adult heart.

Additional AS events identified in our transcriptomic analysis and supported by isoform-specific peptide evidence are summarized in Table S8.

### A subset of splicing events distinguishes iPSC-CMs from hearts

**Figure 6:**
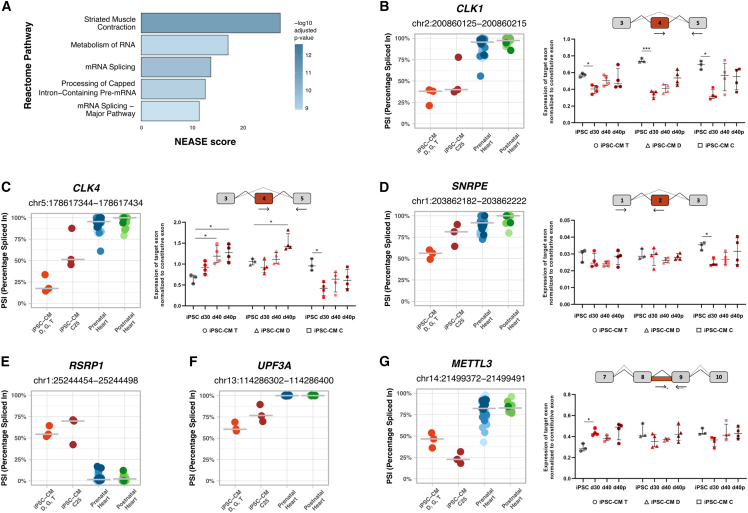
Splicing events with divergent patterns in iPSC-CMs and hearts (A) Top 5 enriched Reactome pathways identified by NEASE for differentially spliced exons identified between iPSC-CMs and both prenatal and postnatal hearts. (B–G) Inclusion levels (PSI) of exons/introns differentially spliced between iPSC-CMs and both prenatal and postnatal hearts, specifically (B) *CLK1* exon 4 (*CLK1*-201), (C) *CLK4* exon 4 (*CLK4*-201), (D) *SNRPE* exon 2 (*SNRPE*-202), (E) *RSRP1* exon 4 (*RSRP1*-*204)*, (F) *UPF3A* exon 4 (*UPF3A*-202), and (G) *METTL3* intron 8 (*METTL3*-201). PSI levels are shown for iPSC-CMs purified by fluorescence-activated cell sorting with VCAM1 antibodies (iPSC-CMs D, G, and T), iPSC-CMs purified by metabolic selection with lactate (iPSC-CM C25), prenatal hearts, and postnatal hearts. (B), (C), (D), and (G) are accompanied by *in vitro* PCR quantification performed in three distinct cell lines (T, D, and C) under four experimental conditions: iPSC not differentiated (in gray), 30 days of differentiation (in red), 40 days of differentiation (in pink), and 40 days of differentiation with micropatterning (in dark red). Significance levels are indicated as follows: ∗(*p* < 0.05) and ∗∗∗(*p* < 0.001).

In addition to *CAMK2D* exon 18, whose inclusion is developmentally regulated in the heart but remains largely unchanged during iPSC-CM maturation (Figure 5G), we identified a subset of splicing events that display distinct patterns in iPSC-CMs compared to human hearts, irrespective of developmental stage (Figure 1J, clusters 2 and 3; Figure S1N, cluster 2). Our statistical analysis revealed 333 exons with splicing patterns unique to iPSC-CMs (Table S9). These events were enriched in genes associated with striated muscle contraction and RNA metabolism, including mRNA splicing (Figure 6A; Table S10).

Many splicing events that differ between iPSC-CMs and heart tissue occur in genes not exclusively expressed in cardiomyocytes, raising the possibility that differences in cell-type composition could confound interpretation. In heart samples, however, these events were typically constitutively spliced, with PSI values approaching 0 or 1, indicating near-complete exon exclusion or inclusion across developmental stages (Figures 6B–6G). In contrast, iPSC-CMs displayed intermediate PSI values for the same events, reflecting greater splicing variability (Figures 6B–6G). These results suggest that the observed splicing differences in iPSC-CMs are unlikely to result from cellular heterogeneity but rather represent intrinsic features of iPSC-CM biology or the *in vitro* differentiation environment, which diverges from the splicing regulation observed in native heart tissue.

To assess whether iPSC-CM-specific splicing events could result from variability introduced by the differentiation protocol, we expanded our analysis to include RNA-seq datasets from iPSC-derived cultures enriched for cardiomyocytes by metabolic selection with lactate (iPSC-CM C25), in addition to cultures purified by fluorescence-activated cell sorting with VCAM1 antibodies (iPSC-CM C, D, G, T). Overall, iPSC-CMs generated with either protocol displayed similar splicing patterns across developmentally regulated events in cardiomyocyte-enriched genes (Figures S6A–S6F). A few exceptions were observed, with metabolically selected iPSC-CMs showing PSI values shifted toward either earlier (Figures S6G and S6H) or later (Figures S6I and S6J) developmental stages relative to antibody-sorted iPSC-CMs.

For iPSC-CM-specific splicing events, both protocols consistently yielded intermediate PSI values for exons that are constitutively spliced in native heart tissue (Figures 6B–6G), indicating that the splicing differences observed in iPSC-CMs are not attributable to the differentiation method but rather represent shared features of *in vitro* derived cardiomyocytes.

For instance, exon 4 of *CLK1* and *CLK4* was more frequently skipped in iPSC-CMs from both protocols compared to heart tissue (Figures 6B and 6C). Exon 4 skipping produces truncated, catalytically inactive isoforms of these kinases (Colwill et al., 1996). A trend toward increased exon 4 inclusion was observed during iPSC-CM maturation (Figures 6B and 6C), and significant changes were detected between iPSCs and iPSC-CMs (Figures 6B and 6C).

Increased skipping of *SNRPE* exon 2, which encodes part of the LSM domain required for spliceosome assembly, was also observed in iPSC-CMs relative to heart tissue, regardless of protocol (Figure 6D). Unlike *CLK1/4*, this exon was not dynamically regulated during iPSC differentiation or iPSC-CM maturation.

Conversely, *RSRP1* exon 4 showed higher inclusion in iPSC-CMs than in heart tissue, regardless of protocol (Figure 6E). Inclusion of this exon disrupts the open reading frame and is predicted to trigger NMD, consistent with lower *RSRP1* expression levels in iPSC-CMs (Figure S6K).

Beyond splicing regulators, iPSC-CM-specific patterns were also observed in genes linked to mRNA decay and modification. Inclusion of *UPF3A* exon 4, which encodes a domain required for NMD machinery interactions, was consistently reduced in iPSC-CMs compared to heart tissue (Figure 6F). In the m6A methyltransferase *METTL3*, intron 8 retention, which disrupts the methylation domain and produces a truncated, non-functional isoform, was consistently lower in iPSC-CMs (Figure 6G). Notably, *METTL3* splicing remained largely unchanged during iPSC differentiation and iPSC-CM maturation (Figure 6G).

Finally, we observed differences in contractile gene splicing. In heart tissue, inclusion of *TPM1* exon 2, which encodes part of the tropomyosin domain, exceeded 94%, whereas inclusion was approximately 75% in iPSC-CMs D, G, and T and ∼88% in iPSC-CMs C25 (Figure S6L).

In summary, we identified a subset of unique splicing events that distinguish iPSC-CMs from native cardiac tissue. Despite some protocol-driven variability, most of the splicing patterns were consistent across iPSC-CMs generated with distinct differentiation methods.

Having identified splicing alterations in iPSC-CMs, we next investigated whether changes in the expression of *trans*-acting splicing regulators could underlie these differences. Our analysis revealed that several mRNAs encoding splicing factors were significantly downregulated in iPSC-CMs compared to pre- and postnatal hearts. These included SR protein kinases CLK1 and SRPK3, as well as ELAVL3, ELAVL4, and NOVA2 (Table S11). Cross-referencing with single-cell transcriptomic data from the fetal heart atlas (Farah et al., 2024) showed that most of these genes are predominantly expressed in non-cardiomyocyte cell types. However, *SRPK3* is specifically expressed in cardiomyocytes (Table S7), raising the possibility that its reduced expression in iPSC-CMs (Figure S6M) may contribute to the altered splicing landscape observed *in vitro*.

## Discussion

In this study, we present a comprehensive characterization of AS dynamics during cardiomyocyte development *in vivo* and *in vitro*, and we highlight discrepancies in splicing regulation between iPSC-CMs and human hearts.

During pre- and postnatal development of the heart, cardiomyocytes undergo a maturation process that involves a wide spectrum of changes in cell structure, metabolism, function, and gene expression (Guo and Pu, 2020). Our comparative analysis of gene expression profiles showed that iPSC-CMs cluster more closely with prenatal heart tissue than postnatal hearts, in agreement with previous reports (Synnergren et al., 2012; Van Den Berg et al., 2015).

AS is a critical post-transcriptional mechanism that regulates gene expression, affecting over 90% of human genes and allowing a single locus to generate multiple transcript variants with diverse properties (Nilsen and Graveley, 2010; Wang et al., 2008). In the heart, developmentally regulated AS has been implicated in the extensive remodeling required to accommodate the increased workload during the transition from fetal to neonatal life and continuing into adulthood (Giudice and Cooper, 2014; Li et al., 2024; Weeland et al., 2015). One well-characterized example is the embryonic-specific EH-myomesin isoform, generated by inclusion of *MYOM1* exon 18. This exon encodes a protein domain that enhances elasticity and modulates the viscoelastic proprieties of the sarcomere (Agarkova et al., 2000).

Despite recent progress, a comprehensive understanding of splicing regulation throughout human heart development remains incomplete. In our analysis, we identified the majority of alternative splicing events previously well characterized as regulated during cardiac development *in vivo*. Furthermore, we show that splicing isoforms reported in the developing murine heart are conserved in the human heart (Table S4), and we discovered novel splicing events that are differentially regulated between pre- and postnatal human hearts (Table S4). While some of these events have been described in the context of disease, their regulation during normal heart development was previously unknown.

In addition to splicing differences between pre- and postnatal hearts, we identified novel splicing events that undergo regulation during prenatal life, between embryonic and fetal stages (Tables S5 and S6).

Taken together, our findings offer a detailed perspective on the temporal dynamics of splicing switches during human heart development, uncovering previously uncharted splicing programs associated with cardiac maturation. Importantly, our analysis shows that iPSC-CMs do not correspond to prenatal hearts of a specific gestational age based on their splicing profiles. Instead, splicing patterns in iPSC-CMs are heterogeneous: some events resemble those of early embryonic hearts (Figures 4C–4F and S4B–S4D), while others align more closely with later fetal stages (Figures 4G and S4E–S4G) or display intermediate profiles (Figures 4H–4J). This variability highlights the heterogeneous maturation state of iPSC-CMs and reflects their incomplete recapitulation of *in vivo* cardiac development.

Additionally, we identified a subset of splicing events that are misregulated in iPSC-CMs, with inclusion levels deviating from the constitutive patterns seen *in vivo* (Tables S9 and S10). Notably, these iPSC-CM-specific splicing alterations include the mis-splicing of splicing regulators as well as factors involved in mRNA decay and modification. These combined splicing defects suggest widespread disruptions in RNA homeostasis, which may contribute to the functional immaturity of iPSC-CMs compared to native heart cells.

Multiple splicing factors have been implicated in the regulation of cardiac-specific splicing programs. Namely, CELF and MBNL proteins are responsible for many splicing transitions that occur during postnatal heart development in mice (Kalsotra et al., 2008; Wang et al., 2012), while SRSF1 is critical role for maintaining splicing patterns during postnatal cardiac remodeling (Xu et al., 2005). Additional RNA-binding proteins involved in cardiac splicing regulation include *PTBP1* (Cao et al., 2021; Charlet-B et al., 2002), *RBFOX2* (Verma et al., 2022), *SRSF5* (Zhang et al., 2021), *SRSF10* (Feng et al., 2009), *RBM24* (Poon et al., 2012; Yang et al., 2014), *RBM20* (Guo et al., 2012; Li et al., 2013), and *QKI* (Montañés-Agudo et al., 2022). None of the transcripts encoding these proteins were differentially expressed in iPSC-CMs compared to heart tissue (Table S11). However, we found significantly reduced expression of *CLK1* and *SRPK3* in iPSC-CMs (Table S11), both of which encode kinases that phosphorylate the serine/arginine-rich (SR) domains of splicing factors (Colwill et al., 1996; Zhong et al., 2009). *SRPK3* is of particular interest because it is specifically expressed in cardiomyocytes and phosphorylates RBM20, a key cardiac splicing regulator (Töpf et al., 2024). Given that RBM20 activity and nuclear localization depend on its phosphorylation status (Murayama et al., 2018), reduced *SRPK3* expression in iPSC-CMs may impair RBM20 function, thus contributing to the altered splicing landscape observed *in vitro*. Importantly, such phosphorylation defects could also help explain the persistence of fetal-like isoforms in iPSC-CMs, as several splicing factors critical for adult splicing programs may remain in a functionally inactive state.

In contrast to *SRPK3*, *CLK1*, another kinase known to phosphorylate RBM20 (Sun et al., 2022), is ubiquitously expressed in the heart. Its downregulation in iPSC-CMs may reflect the absence of non-cardiomyocyte cell types. However, we also observed skipping of the exon encoding the *CLK1* kinase domain in iPSC-CMs, a change likely to impair its enzymatic function. Taken together, these findings support a model in which defective phosphorylation driven by reduced *SRPK3* expression and altered *CLK1* splicing acts upstream of the splicing differences observed in iPSC-CMs.

In conclusion, our findings highlight the ability of iPSC-CMs to model key aspects of early heart development, while also revealing their limitations in recapitulating the full spectrum of splicing programs that govern cardiac maturation. The observed splicing immaturity and variability in iPSC-CMs underscore the need for further optimization of maturation protocols to more faithfully replicate native cardiac splicing transitions, ultimately enhancing their utility as robust *in vitro* models for cardiovascular research.

## Methods

### Human iPSCs

In this study, the following human iPSC lines were used. The DF6.9.9 T.B cell line, provided by the WiCell Bank (https://www.wicell.org/), was reprogrammed from male foreskin fibroblasts (Yu et al., 2009) and is here referred to as iPSC-D. The Gibco Human Episomal iPSC line (iPSC6.2), here referred to as iPSC-G, was derived from female CD34^+^ cord blood cells (Burridge et al., 2014). The Cuso-2 iPSC line, here referred as iPSC-C, was reprogrammed from skin fibroblasts of a healthy male donor (Knight et al., 2021). The cell line F002.1A.13, here referred to as iPSC-T, was derived from skin fibroblasts of a healthy female donor (Silva et al., 2020). The iPSC line BIHi005-A, here referred as C25, was a gift from A. Moretti, Munich, Germany (Moretti et al., 2010).

### Cardiomyocyte differentiation

Cardiomyocytes differentiated from iPSC lines D, G, T, and C were generated as previously described (Jager et al., 2024). On day 13 of differentiation, cellular aggregates were dissociated using 0.25% Trypsin-EDTA (Gibco) for 7 min at 37°C. After dissociation, cells were washed with 2% fetal bovine serum (FBS) and 2 mM EDTA in phosphate-buffered saline (PBS, 0.1M) and incubated with VCAM1 antibody (BioLegend, 1:50) diluted in PBS/2% FBS for 15 min at 4°C. Then, cells were washed and suspended in PBS/2% FBS for FACS sorting (BD FACSAria III Cell Sorter) using a 100 μm nozzle at 4°C. Purified VCAM1-positive cells were plated on wells coated with Matrigel, at a seeding density between 20,000 and 40,000 cells/cm^2^. Alternatively, cardiomyocytes were differentiated from the iPSC C25 line, as described (Breckwoldt et al., 2017; Radke et al., 2021).

### iPSC-CM culture on a micropatterned surface

On day 30 of differentiation, iPSC-CMs were replaced by incubating with 0.25% Trypsin-EDTA (Gibco) for 3 min at 37°C, followed by trypsin neutralization using PBS supplemented with 10% FBS. Cells were cultured on micropatterned coverslips (4Dcell, 10 mm × 10 mm, 100 μm pattern) for an additional 10 days. The RPMI + B27 medium was replaced every 2 days, and at day 40 of differentiation coverslips were fixed in 3.7% PFA and iPSC-CMs were collected in NZYol (NZYTech) for subsequent RNA analysis.

### Fluorescence microscopy

Immunofluorescence was performed as previously described (Jager et al., 2024) using a mouse anti-myosin-binding protein C antibody (Santa Cruz Biotechnology, sc-131781), a rabbit anti-α-actinin antibody (Abcam, ab9465) and Alexa Fluor 546 phalloidin for actin staining. Fluorescence images were acquired with Zeiss LSM 710 Confocal Laser Point-Scanning Microscope.

### Quantitative RT-PCR

Total RNA was extracted using NZYol (NZYTech), and cDNA synthesis was performed using the SuperScript IV Reverse Transcriptase (Invitrogen). RT-qPCR was carried out using the Universal SYBR Green Supermix (Bio-Rad). Detailed procedures and primer sequences are provided in the supplemental information.

### Single-cell RNA sequencing

Single-cell transcriptomes of day 30 iPSC-derived cardiomyocytes were generated using the Chromium Single Cell 3ʹ GEM, Library & Gel Bead Kit v3 (10× Genomics, PN-1000092). Libraries were sequenced on an Illumina NextSeq 500 platform. Full experimental details and data processing steps are described in the supplemental information.

### Bulk RNA-sequencing

RNA from iPSC-CMs at day 30 of differentiation was collected and sequenced on the Illumina platform Novaseq6000 platform. Reads were mapped to the human reference genome (GRCh38/hg38) using *STAR v.2.7.10b* (Dobin et al., 2013). AS events were identified and quantified using *rMATS v4.1.2*, *MAJIQ v.2.5.1* and *vast-tools v.2.5.1* (Shen et al., 2014; Tapial et al., 2017; Vaquero-Garcia et al., 2023). Functional enrichment of AS events was assessed with *NEASE v.1.2.2* (Louadi et al., 2021). A full description of the methods and parameter settings is provided in the supplemental information.

### Mass spectrometry

iPSC-CMs at day 30 of differentiation were collected and multiplexed using TMTpro 16-plex reagents. LC-MS/MS was performed on an Orbitrap Fusion Lumos mass spectrometer in data-dependent acquisition mode. Protein identification and quantification were carried out using MaxQuant v.2.6.4.0. Comprehensive information on sample preparation, acquisition, and data analysis is available in the supplemental information.

## Resource availability

### Lead contact

Requests for further information should be directed to and will be fulfilled by the lead contact, M. Carmo-Fonseca (carmo.fonseca@gimm.pt).

### Materials availability

The materials used in this study are available from the corresponding author upon request.

### Data and code availability

• Raw single-cell RNA sequencing (scRNA-seq) data from iPSC-CMs are available under accession number E-MTAB-13850. Bulk RNA sequencing raw FASTQ files are available in E-MTAB-13757. The LC-MS/MS proteomic data generated from iPSC-CMs in this study have been deposited to the ProteomeXchange Consortium via the PRIDE (Perez-Riverol et al., 2025) partner repository with the dataset identifier PXD070546 and https://doi.org/10.6019/PXD070546.
• All scripts and code used for bioinformatic analyses are available at https://github.com/bdgsilva/Cardiac-splicing-in-vivo-and-in-vitro.

## Acknowledgments

We are grateful to Leslie Leinwand (University of Colorado, Boulder, CO, USA) and Lars Steinmetz (Stanford University, Palo Alto, CA, USA) for insightful discussions. We also thank the GIMM Flow Cytometry, Bioimaging and Genomics Facilities (Lisboa, Portugal), the BIH/MDC Genomics Technology Platform (Berlin, Germany), and Carmen Judis (MDC, Berlin, Germany) for technical support. Mass-spectrometry-based proteomics services were provided by the VIB Proteomics Core (Ghent, Belgium), whose support is gratefully acknowledged.

This work was supported by la Caixa Foundation under the agreement LCF/PR/HR20/52400021, 10.13039/501100001674Leducq Foundation for Cardiovascular Research network research grant 21CVD02, and 10.13039/100032285Novo Nordisk Foundation (23OC0081287). Further support was received from 10.13039/501100001871Fundação para a Ciência e a Tecnologia (FCT), Portugal (Fellowship 2020.04836 BD to M.F.).

## Author contributions

B.G.-S., M.F., and M.C.-F. designed the study. B.G.-S. performed the bioinformatics analyses. M.F., M.R., S.M., and T.C. performed the experiments involving iPSC-CM differentiation and characterization. A.V.-G. performed the proteomic analysis. P.P. and C.C. contributed RNA-seq data from iPSC-CM line BIHi005-A. H.M. contributed to scRNA-seq. R.S. and M.G. provided expert guidance and feedback on analyses and results. B.G.-S., M.F., and M.C.-F. wrote the manuscript, with feedback from all authors.

## Declaration of interests

The authors M.R., S.M., M.F., T.C., and M.C.-F. filed a patent application covering the cardiomyocyte differentiation protocol presented in this study (Methods of Cardiomyocyte Production, PCT/EP2022/084633, Priority date of December 7, 2021, currently active in Portugal under n° 2023104813). M.G. is an advisor for River Biomedics and collaborates with Ionis Pharmaceuticals. The remaining authors have nothing to disclose.

## Supplemental information

Document S1. Figures S1–S6, Tables S1, S4, S6, S8, S10, S11, S12, and supplemental methods

Table S2. Differentially spliced events detected between prenatal and postnatal hearts

Table S3. Differentially spliced events detected between iPSC-CMs and prenatal hearts and between iPSC-CMs and postnatal hearts

Table S5. Differentially spliced events detected between embryonic and fetal hearts

Table S7. Cardiomyocyte-enriched genes (genes with log2FC > 0.5 when comparing cardiomyocytes with all other cell types in the fetal heart single-cell atlas)

Table S9. Splicing events with divergent patterns in iPSC-CMs and hearts

Document S2. Article plus supplemental information

## References

[bib1] Agarkova I., Auerbach D., Ehler E., Perriard J.C. (2000). A novel marker for vertebrate embryonic heart, the EH-myomesin isoform. J. Biol. Chem..

[bib2] Baralle F.E., Giudice J. (2017). Alternative splicing as a regulator of development and tissue identity. Nat. Rev. Mol. Cell Biol..

[bib3] Blencowe B.J. (2006). Alternative Splicing: New Insights from Global Analyses. Cell.

[bib4] Bock-Bierbaum T., Funck K., Wollweber F., Lisicki E., Von Der Malsburg K., Von Der Malsburg A., Laborenz J., Noel J.K., Hessenberger M., Jungbluth S. (2022). Structural insights into crista junction formation by the Mic60-Mic19 complex. Sci. Adv..

[bib5] Braunschweig U., Barbosa-Morais N.L., Pan Q., Nachman E.N., Alipanahi B., Gonatopoulos-Pournatzis T., Frey B., Irimia M., Blencowe B.J. (2014). Widespread intron retention in mammals functionally tunes transcriptomes. Genome Res..

[bib6] Breckwoldt K., Letuffe-Brenière D., Mannhardt I., Schulze T., Ulmer B., Werner T., Benzin A., Klampe B., Reinsch M.C., Laufer S. (2017). Differentiation of cardiomyocytes and generation of human engineered heart tissue. Nat. Protoc..

[bib7] Buijtendijk M.F.J., Barnett P., Van Den Hoff M.J.B. (2020). Development of the human heart. Am. J. Med. Genet. C.

[bib8] Burridge P.W., Matsa E., Shukla P., Lin Z.C., Churko J.M., Ebert A.D., Lan F., Diecke S., Huber B., Mordwinkin N.M. (2014). Chemically defined generation of human cardiomyocytes. Nat. Methods.

[bib9] Burridge P.W., Sharma A., Wu J.C. (2015). Genetic and Epigenetic Regulation of Human Cardiac Reprogramming and Differentiation in Regenerative Medicine. Annu. Rev. Genet..

[bib10] Cao J., O’Day D.R., Pliner H.A., Kingsley P.D., Deng M., Daza R.M., Zager M.A., Aldinger K.A., Blecher-Gonen R., Zhang F. (2020). A human cell atlas of fetal gene expression. Science.

[bib11] Cao J., Routh A.L., Kuyumcu-Martinez M.N. (2021). Nanopore sequencing reveals full-length Tropomyosin 1 isoforms and their regulation by RNA-binding proteins during rat heart development. J. Cell Mol. Med..

[bib12] Cardoso-Moreira M., Halbert J., Valloton D., Velten B., Chen C., Shao Y., Liechti A., Ascenção K., Rummel C., Ovchinnikova S. (2019). Gene expression across mammalian organ development. Nature.

[bib13] Charlet-B N., Logan P., Singh G., Cooper T.A. (2002). Dynamic Antagonism between ETR-3 and PTB Regulates Cell Type-Specific Alternative Splicing. Mol. Cell.

[bib14] Chopra N., Knollmann B.C. (2013). Triadin regulates cardiac muscle couplon structure and microdomain Ca2+ signalling: a path towards ventricular arrhythmias. Cardiovasc. Res..

[bib15] Christofk H.R., Vander Heiden M.G., Harris M.H., Ramanathan A., Gerszten R.E., Wei R., Fleming M.D., Schreiber S.L., Cantley L.C. (2008). The M2 splice isoform of pyruvate kinase is important for cancer metabolism and tumour growth. Nature.

[bib16] Colwill K., Pawson T., Andrews B., Prasad J., Manley J.L., Bell J.C., Duncan P.I. (1996). The Clk/Sty protein kinase phosphorylates SR splicing factors and regulates their intranuclear distribution. EMBO J..

[bib17] Cui Y., Zheng Y., Liu X., Yan L., Fan X., Yong J., Hu Y., Dong J., Li Q., Wu X. (2019). Single-Cell Transcriptome Analysis Maps the Developmental Track of the Human Heart. Cell Rep..

[bib18] Di Minin G., Holzner M., Grison A., Dumeau C.E., Chan W., Monfort A., Jerome-Majewska L.A., Roelink H., Wutz A. (2022). TMED2 binding restricts SMO to the ER and Golgi compartments. PLoS Biol..

[bib19] Dobin A., Davis C.A., Schlesinger F., Drenkow J., Zaleski C., Jha S., Batut P., Chaisson M., Gingeras T.R. (2013). STAR: ultrafast universal RNA-seq aligner. Bioinformatics.

[bib20] Doll S., Dreßen M., Geyer P.E., Itzhak D.N., Braun C., Doppler S.A., Meier F., Deutsch M.-A., Lahm H., Lange R. (2017). Region and cell-type resolved quantitative proteomic map of the human heart. Nat. Commun..

[bib21] Duran J., Nickel L., Estrada M., Backs J., Van Den Hoogenhof M.M.G. (2021). CaMKIIδ Splice Variants in the Healthy and Diseased Heart. Front. Cell Dev. Biol..

[bib22] Efthymiou G., Radwanska A., Grapa A.-I., Beghelli-de La Forest Divonne S., Grall D., Schaub S., Hattab M., Pisano S., Poet M., Pisani D.F. (2021). Fibronectin Extra Domains tune cellular responses and confer topographically distinct features to fibril networks. J. Cell Sci..

[bib23] Farah E.N., Hu R.K., Kern C., Zhang Q., Lu T.-Y., Ma Q., Tran S., Zhang B., Carlin D., Monell A. (2024). Spatially organized cellular communities form the developing human heart. Nature.

[bib24] Feng Y., Valley M.T., Lazar J., Yang A.L., Bronson R.T., Firestein S., Coetzee W.A., Manley J.L. (2009). SRp38 regulates alternative splicing and is required for Ca(2+) handling in the embryonic heart. Dev. Cell.

[bib25] Galdos F.X., Lee C., Lee S., Paige S., Goodyer W., Xu S., Samad T., Escobar G.V., Darsha A., Beck A. (2023). Combined lineage tracing and scRNA-seq reveals unexpected first heart field predominance of human iPSC differentiation. eLife.

[bib26] Giudice J., Cooper T.A., Yeo G.W. (2014). Systems Biology of RNA Binding Proteins.

[bib35] Giudice J., Xia Z., Wang E.T., Scavuzzo M.A., Ward A.J., Kalsotra A., Wang W., Wehrens X.H.T., Burge C.B., Li W., Cooper T.A. (2014). Alternative splicing regulates vesicular trafficking genes in cardiomyocytes during postnatal heart development. Nat. Commun..

[bib27] Godbout E., Son D.O., Hume S., Boo S., Sarrazy V., Clément S., Kapus A., Wehrle-Haller B., Bruckner-Tuderman L., Has C., Hinz B. (2020). Kindlin-2 Mediates Mechanical Activation of Cardiac Myofibroblasts. Cells.

[bib28] Gomes A.V., Guzman G., Zhao J., Potter J.D. (2002). Cardiac troponin T isoforms affect the Ca2+ sensitivity and inhibition of force development. Insights into the role of troponin T isoforms in the heart. J. Biol. Chem..

[bib29] Gönczi M., Teixeira J.M.C., Barrera-Vilarmau S., Mediani L., Antoniani F., Nagy T.M., Fehér K., Ráduly Z., Ambrus V., Tőzsér J. (2023). Alternatively spliced exon regulates context-dependent MEF2D higher-order assembly during myogenesis. Nat. Commun..

[bib30] Guo Y., Pu W.T. (2020). Cardiomyocyte Maturation: New Phase in Development. Circ. Res..

[bib31] Guo W., Schafer S., Greaser M.L., Radke M.H., Liss M., Govindarajan T., Maatz H., Schulz H., Li S., Parrish A.M. (2012). RBM20, a gene for hereditary cardiomyopathy, regulates titin splicing. Nat. Med..

[bib32] Huang C., Zhou Q., Liang P., Hollander M.S., Sheikh F., Li X., Greaser M., Shelton G.D., Evans S., Chen J. (2003). Characterization and in Vivo Functional Analysis of Splice Variants of Cypher. J. Biol. Chem..

[bib33] Jager J., Ribeiro M., Furtado M., Carvalho T., Syrris P., Lopes L.R., Elliott P.M., Cabral J.M.S., Carmo-Fonseca M., Da Rocha S.T., Martins S. (2024). Patient-derived induced pluripotent stem cells to study non-canonical splicing variants associated with Hypertrophic Cardiomyopathy. Stem Cell Res..

[bib34] Jiang X., Lian X., Wei K., Zhang J., Yu K., Li H., Ma H., Cai Y., Pang L. (2024). Maturation of pluripotent stem cell-derived cardiomyocytes: limitations and challenges from metabolic aspects. Stem Cell Res. Ther..

[bib36] John G.B., Shang Y., Li L., Renken C., Mannella C.A., Selker J.M.L., Rangell L., Bennett M.J., Zha J. (2005). The Mitochondrial Inner Membrane Protein Mitofilin Controls Cristae Morphology. MBoC.

[bib37] Kalsotra A., Xiao X., Ward A.J., Castle J.C., Johnson J.M., Burge C.B., Cooper T.A. (2008). A postnatal switch of CELF and MBNL proteins reprograms alternative splicing in the developing heart. Proc. Natl. Acad. Sci. USA.

[bib38] Karbassi E., Fenix A., Marchiano S., Muraoka N., Nakamura K., Yang X., Murry C.E. (2020). Cardiomyocyte maturation: advances in knowledge and implications for regenerative medicine. Nat. Rev. Cardiol..

[bib39] Kjer-Hansen P., Weatheritt R.J. (2023). The function of alternative splicing in the proteome: rewiring protein interactomes to put old functions into new contexts. Nat. Struct. Mol. Biol..

[bib40] Knight W.E., Cao Y., Lin Y.-H., Chi C., Bai B., Sparagna G.C., Zhao Y., Du Y., Londono P., Reisz J.A. (2021). Maturation of Pluripotent Stem Cell-Derived Cardiomyocytes Enables Modeling of Human Hypertrophic Cardiomyopathy. Stem Cell Rep..

[bib41] Li S., Guo W., Dewey C.N., Greaser M.L. (2013). Rbm20 regulates titin alternative splicing as a splicing repressor. Nucleic Acids Res..

[bib42] Li Z., Cao C., Zhao Q., Li D., Han Y., Zhang M., Mao L., Zhou B., Wang L. (2025). RNA splicing controls organ-wide maturation of postnatal heart in mice. Dev. Cell.

[bib43] Louadi Z., Elkjaer M.L., Klug M., Lio C.T., Fenn A., Illes Z., Bongiovanni D., Baumbach J., Kacprowski T., List M., Tsoy O. (2021). Functional enrichment of alternative splicing events with NEASE reveals insights into tissue identity and diseases. Genome Biol..

[bib44] Lu F., Ma Q., Xie W., Liou C.L., Zhang D., Sweat M.E., Jardin B.D., Naya F.J., Guo Y., Cheng H., Pu W.T. (2022). CMYA5 establishes cardiac dyad architecture and positioning. Nat. Commun..

[bib45] Luna-Zurita L., Stirnimann C.U., Glatt S., Kaynak B.L., Thomas S., Baudin F., Samee M.A.H., He D., Small E.M., Mileikovsky M. (2016). Complex Interdependence Regulates Heterotypic Transcription Factor Distribution and Coordinates Cardiogenesis. Cell.

[bib46] Maroli G., Braun T. (2021). The long and winding road of cardiomyocyte maturation. Cardiovasc. Res..

[bib47] Mazin P.V., Khaitovich P., Cardoso-Moreira M., Kaessmann H. (2021). Alternative splicing during mammalian organ development. Nat. Genet..

[bib48] Meilhac S.M., Buckingham M.E. (2018). The deployment of cell lineages that form the mammalian heart. Nat. Rev. Cardiol..

[bib49] Merkin J., Russell C., Chen P., Burge C.B. (2012). Evolutionary Dynamics of Gene and Isoform Regulation in Mammalian Tissues. Science.

[bib50] Montañés-Agudo P., Aufiero S., Schepers E.N., van der Made I., Pinto Y.M., Creemers E. (2022). The RNA-binding protein QKI governs a muscle-specific alternative splicing program that shapes the contractile function of cardiomyocytes. J. Mol. Cell. Cardiol..

[bib51] Moretti A., Bellin M., Welling A., Jung C.B., Lam J.T., Bott-Flügel L., Dorn T., Goedel A., Höhnke C., Hofmann F. (2010). Patient-Specific Induced Pluripotent Stem-Cell Models for Long-QT Syndrome. N. Engl. J. Med..

[bib52] Murayama R., Kimura-Asami M., Togo-Ohno M., Yamasaki-Kato Y., Naruse T.K., Yamamoto T., Hayashi T., Ai T., Spoonamore K.G., Kovacs R.J. (2018). Phosphorylation of the RSRSP stretch is critical for splicing regulation by RNA-Binding Motif Protein 20 (RBM20) through nuclear localization. Sci. Rep..

[bib53] Nilsen T.W., Graveley B.R. (2010). Expansion of the eukaryotic proteome by alternative splicing. Nature.

[bib54] Olson E.N. (2006). Gene Regulatory Networks in the Evolution and Development of the Heart. Science.

[bib85] Perez-Riverol Y., Bandla C., Kundu J.D., Kamatchinathan S., Bai J., Hewapathirana S., John S.D. (2025). The PRIDE database at 20 years: 2025 update. Nucleic Acid Res.

[bib55] Poon K.L., Tan K.T., Wei Y.Y., Ng C.P., Colman A., Korzh V., Xu X.Q. (2012). RNA-binding protein RBM24 is required for sarcomere assembly and heart contractility. Cardiovasc. Res..

[bib56] Radke M.H., Badillo-Lisakowski V., Britto-Borges T., Kubli D.A., Jüttner R., Parakkat P., Carballo J.L., Hüttemeister J., Liss M., Hansen A. (2021). Therapeutic inhibition of RBM20 improves diastolic function in a murine heart failure model and human engineered heart tissue. Sci. Transl. Med..

[bib57] Shen S., Park J.W., Lu Z.x., Lin L., Henry M.D., Wu Y.N., Zhou Q., Xing Y. (2014). rMATS: Robust and flexible detection of differential alternative splicing from replicate RNA-Seq data. Proc. Natl. Acad. Sci. USA.

[bib58] Silva T.P., Bekman E.P., Fernandes T.G., Vaz S.H., Rodrigues C.A.V., Diogo M.M., Cabral J.M.S., Carmo-Fonseca M. (2020). Maturation of Human Pluripotent Stem Cell-Derived -Cerebellar Neurons in the Absence of Co-culture. Front. Bioeng. Biotechnol..

[bib59] Sun M., Jin Y., Zhang Y., Gregorich Z.R., Ren J., Ge Y., Guo W. (2022). SR Protein Kinases Regulate the Splicing of Cardiomyopathy-Relevant Genes via Phosphorylation of the RSRSP Stretch in RBM20. Genes.

[bib60] Sylva M., Van Den Hoff M.J.B., Moorman A.F.M. (2014). Development of the human heart. Am. J. Med. Genet..

[bib61] Synnergren J., Améen C., Jansson A., Sartipy P. (2012). Global transcriptional profiling reveals similarities and differences between human stem cell-derived cardiomyocyte clusters and heart tissue. Physiol. Genom..

[bib62] Takahashi K., Tanabe K., Ohnuki M., Narita M., Ichisaka T., Tomoda K., Yamanaka S. (2007). Induction of Pluripotent Stem Cells from Adult Human Fibroblasts by Defined Factors. Cell.

[bib63] Tapial J., Ha K.C.H., Sterne-Weiler T., Gohr A., Braunschweig U., Hermoso-Pulido A., Quesnel-Vallières M., Permanyer J., Sodaei R., Marquez Y. (2017). An atlas of alternative splicing profiles and functional associations reveals new regulatory programs and genes that simultaneously express multiple major isoforms. Genome Res..

[bib64] Timpl R., Sasaki T., Kostka G., Chu M.-L. (2003). Fibulins: a versatile family of extracellular matrix proteins. Nat. Rev. Mol. Cell Biol..

[bib65] Töpf A., Cox D., Zaharieva I.T., Di Leo V., Sarparanta J., Jonson P.H., Sealy I.M., Smolnikov A., White R.J., Vihola A. (2024). Digenic inheritance involving a muscle-specific protein kinase and the giant titin protein causes a skeletal muscle myopathy. Nat. Genet..

[bib66] Tyser R.C.V., Ibarra-Soria X., McDole K., Arcot Jayaram S., Godwin J., Van Den Brand T.A.H., Miranda A.M.A., Scialdone A., Keller P.J., Marioni J.C., Srinivas S. (2021). Characterization of a common progenitor pool of the epicardium and myocardium. Science.

[bib67] Van Den Berg C.W., Okawa S., Chuva De Sousa Lopes S.M., Van Iperen L., Passier R., Braam S.R., Tertoolen L.G., Del Sol A., Davis R.P., Mummery C.L. (2015). Transcriptome of human foetal heart compared with cardiomyocytes from pluripotent stem cells. Development.

[bib68] Van Den Hoogenhof M.M.G., Pinto Y.M., Creemers E.E. (2016). RNA Splicing: Regulation and Dysregulation in the Heart. Circ. Res..

[bib69] Vaquero-Garcia J., Aicher J.K., Jewell S., Gazzara M.R., Radens C.M., Jha A., Norton S.S., Lahens N.F., Grant G.R., Barash Y. (2023). RNA splicing analysis using heterogeneous and large RNA-seq datasets. Nat. Commun..

[bib70] Verma S.K., Deshmukh V., Thatcher K., Belanger K.K., Rhyner A.M., Meng S., Holcomb R.J., Bressan M., Martin J.F., Cooke J.P. (2022). RBFOX2 is required for establishing RNA regulatory networks essential for heart development. Nucleic Acids Res..

[bib71] Vučković S., Dinani R., Nollet E.E., Kuster D.W.D., Buikema J.W., Houtkooper R.H., Nabben M., Van Der Velden J., Goversen B. (2022). Characterization of cardiac metabolism in iPSC-derived cardiomyocytes: lessons from maturation and disease modeling. Stem Cell Res. Ther..

[bib72] Wang E.T., Sandberg R., Luo S., Khrebtukova I., Zhang L., Mayr C., Kingsmore S.F., Schroth G.P., Burge C.B. (2008). Alternative isoform regulation in human tissue transcriptomes. Nature.

[bib73] Wang E.T., Cody N.A.L., Jog S., Biancolella M., Wang T.T., Treacy D.J., Luo S., Schroth G.P., Housman D.E., Reddy S. (2012). Transcriptome-wide Regulation of Pre-mRNA Splicing and mRNA Localization by Muscleblind Proteins. Cell.

[bib74] Wang J., An M., Haubner B.J., Penninger J.M. (2022). Cardiac regeneration: Options for repairing the injured heart. Front. Cardiovasc. Med..

[bib75] Weeland C.J., Van Den Hoogenhof M.M., Beqqali A., Creemers E.E. (2015). Insights into alternative splicing of sarcomeric genes in the heart. J. Mol. Cell. Cardiol..

[bib76] Xu X., Yang D., Ding J.-H., Wang W., Chu P.-H., Dalton N.D., Wang H.-Y., Bermingham J.R., Ye Z., Liu F. (2005). ASF/SF2-regulated CaMKIIdelta alternative splicing temporally reprograms excitation-contraction coupling in cardiac muscle. Cell.

[bib77] Yamazaki T., Liu L., Lazarev D., Al-Zain A., Fomin V., Yeung P.L., Chambers S.M., Lu C.-W., Studer L., Manley J.L. (2018). TCF3 alternative splicing controlled by hnRNP H/F regulates E-cadherin expression and hESC pluripotency. Genes Dev..

[bib78] Yang J., Hung L.-H., Licht T., Kostin S., Looso M., Khrameeva E., Bindereif A., Schneider A., Braun T. (2014). RBM24 is a major regulator of muscle-specific alternative splicing. Dev. Cell.

[bib79] Yang X., Coulombe-Huntington J., Kang S., Sheynkman G.M., Hao T., Richardson A., Sun S., Yang F., Shen Y.A., Murray R.R. (2016). Widespread Expansion of Protein Interaction Capabilities by Alternative Splicing. Cell.

[bib80] Yoneda-Kato N., Tomoda K., Umehara M., Arata Y., Kato J.y. (2005). Myeloid leukemia factor 1 regulates p53 by suppressing COP1 via COP9 signalosome subunit 3. EMBO J..

[bib81] Yu J., Hu K., Smuga-Otto K., Tian S., Stewart R., Slukvin I.I., Thomson J.A. (2009). Human Induced Pluripotent Stem Cells Free of Vector and Transgene Sequences. Science.

[bib82] Zaragoza C., Gomez-Guerrero C., Martin-Ventura J.L., Blanco-Colio L., Lavin B., Mallavia B., Tarin C., Mas S., Ortiz A., Egido J. (2011). Animal Models of Cardiovascular Diseases. J. Biomed. Biotechnol..

[bib83] Zhang X., Wang Z., Xu Q., Chen Y., Liu W., Zhong T., Li H., Quan C., Zhang L., Cui C.-P. (2021). Splicing factor Srsf5 deletion disrupts alternative splicing and causes noncompaction of ventricular myocardium. iScience.

[bib84] Zhong X.-Y., Ding J.-H., Adams J.A., Ghosh G., Fu X.-D. (2009). Regulation of SR protein phosphorylation and alternative splicing by modulating kinetic interactions of SRPK1 with molecular chaperones. Genes Dev..

