# Supplementary material for: Alternative splicing dynamics during human cardiac development in vivo and in vitro
Source: Stem Cell Reports. 2026 Jan 2;21(1):102757. doi: 10.1016/j.stemcr.2025.102757 (PMC12925949; doi:10.1016/j.stemcr.2025.102757)
Supplement: Document S1. Figures S1–S6, Tables S1, S4, S6, S8, S10, S11, S12, and supplemental methods [file mmc1.pdf]

Stem Cell Reports, Volume *21*

Supplemental Information

Alternative splicing dynamics during human cardiac development *in vivo* and *in vitro*

Beatriz Gomes-Silva, Marta Furtado, Marta Ribeiro, Sandra Martins, Teresa Carvalho, André Ventura-Gomes, Henrike Maatz, Pragati Parakkat, Claudia Crocini, Michael Gotthardt, Rosina Savisaar, and Maria Carmo-Fonseca

Alternative splicing dynamics during human cardiac development *in vivo* and *in vitro*

Supplementary information

Supplementary Figures + legends

Supplementary Tables

Supplementary Experimental Procedures

Supplementary References

Supplementary Figures + Legends

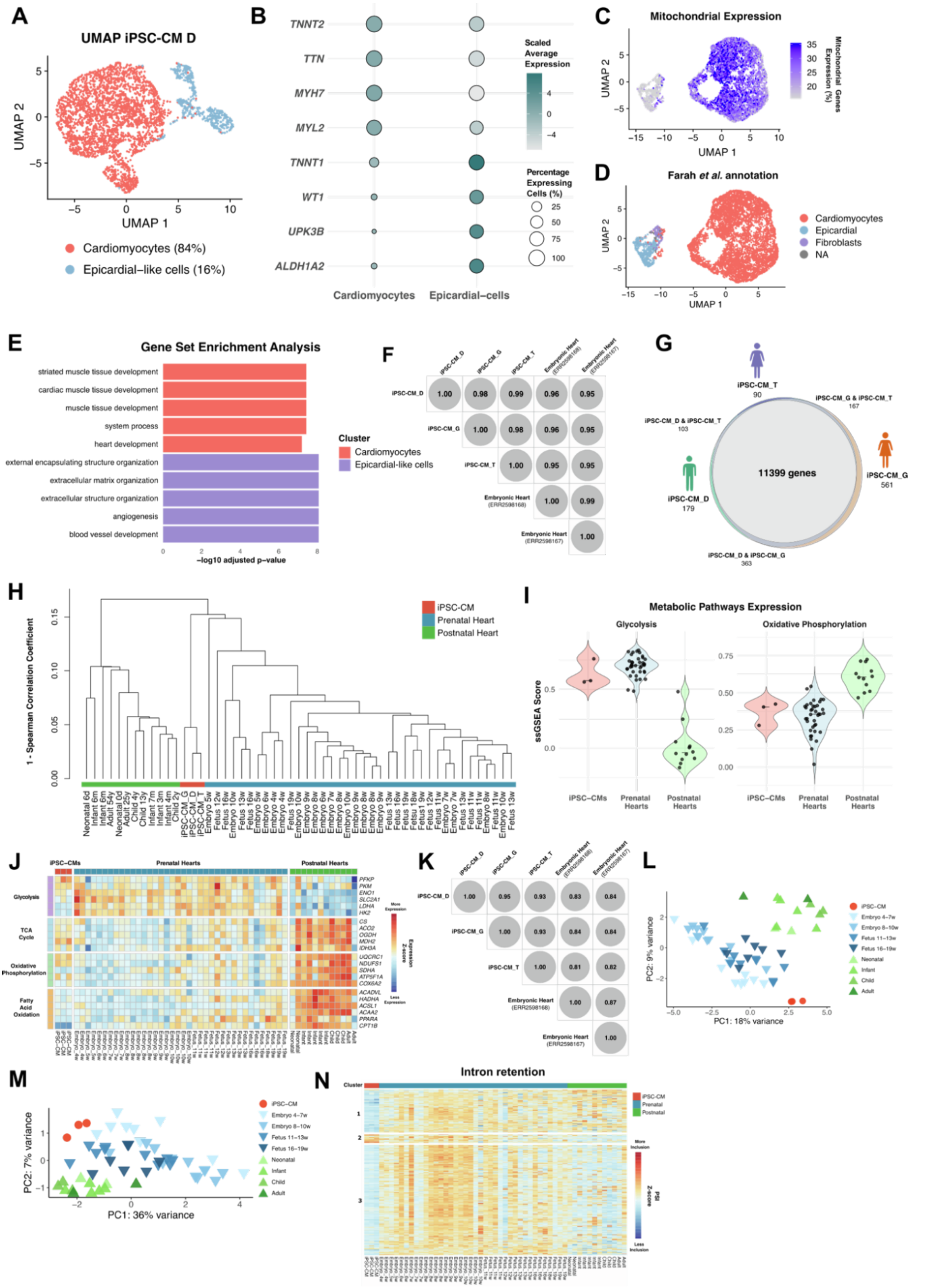

Supplementary Figure 1. Genome-wide comparison of splicing profiles in iPSC-CMs and developing hearts

A. UMAP plot of iPSC-CMs derived from iPSC-D, revealing two major subpopulations of cells corresponding to cardiomyocytes (red), and epicardial-derived cells (blue).

B. Dot plot of the expression of markers for the cell types identified in (**A**). Dot size represents the percentage of cells expressing each gene, and dot color indicates scaled expression levels averaged per cell population.

C. UMAP plots displaying the enriched expression of mitochondrial genes in iPSC-CM derived from iPSC-T.

D. UMAP plot of iPSC-CMs derived from iPSC-T with cell type annotations based on label transfer from the Farah *et al.* single-cell atlas.

E. Gene set enrichment analysis of population-specific markers with adjusted p-value <= 0.01 sorted by average log2FC.

F. Correlation matrix showing gene expression similarities (TPM > 1) between iPSC-CM cultures derived from iPSC-D, iPSC-G, and iPSC-T, and two distinct 4-week embryonic hearts. Gene expression counts were rlog-transformed, and pairwise correlation coefficients were computed using Pearson correlation.

G. Venn diagram illustrating the overlap of the protein-coding genes expressed (TPM>1) in iPSC-CM cultures derived from iPSC-D (green), iPSC-G (orange) and iPSC-T (purple).

H. Hierarchical clustering of all samples, based on 1 – pairwise Spearman correlation coefficients, utilizing the average linkage method. Samples are colored according to their group (iPSC-CMs - red; prenatal heart – blue; postnatal heart - green).

I. Violin plots showing the single-sample GSEA score of iPSC-CMs, prenatal and postnatal hearts for Glycolysis and Oxidative Phosphorylation (OXPHOS) pathways.

J. Heatmap displaying the Z-score normalized expression levels of key genes involved in (from top to bottom) glycolysis, the TCA cycle, oxidative phosphorylation (OXPHOS) and fatty acid ß-oxidation (FAO), across iPSC-CMs, prenatal and postnatal hearts.

K. Correlation matrix showing the inclusion levels (TPM>10) of all alternative splicing events detected by rMATS in iPSC-CM cultures derived from iPSC-D, iPSC-G and iPSC-T. Constitutive events with a Percent Spliced In (PSI) of 0 or 1 were removed. PSIs were logit-transformed and pairwise correlation coefficients were calculated using Spearman correlation.

L-M. PCA of the 500 most variable splicing events in iPSC-CMs (red), prenatal hearts (in blue) and postnatal hearts (in green), as identified by (**L**) MAJIQ and (**M**) rMATS. Lighter colors represent younger samples, while darker colors represent older samples.

N. Heatmap displaying Z-score normalized Percent Spliced In (PSI) levels for all intron retention events, comparing iPSC-CMs with prenatal hearts, iPSC-CMs with postnatal hearts,

and prenatal hearts with postnatal hearts. Colors reflect inclusion relative to the mean of each event (red for higher inclusion, blue for lower inclusion). Event-specific information is provided in Tables S2 and S3.

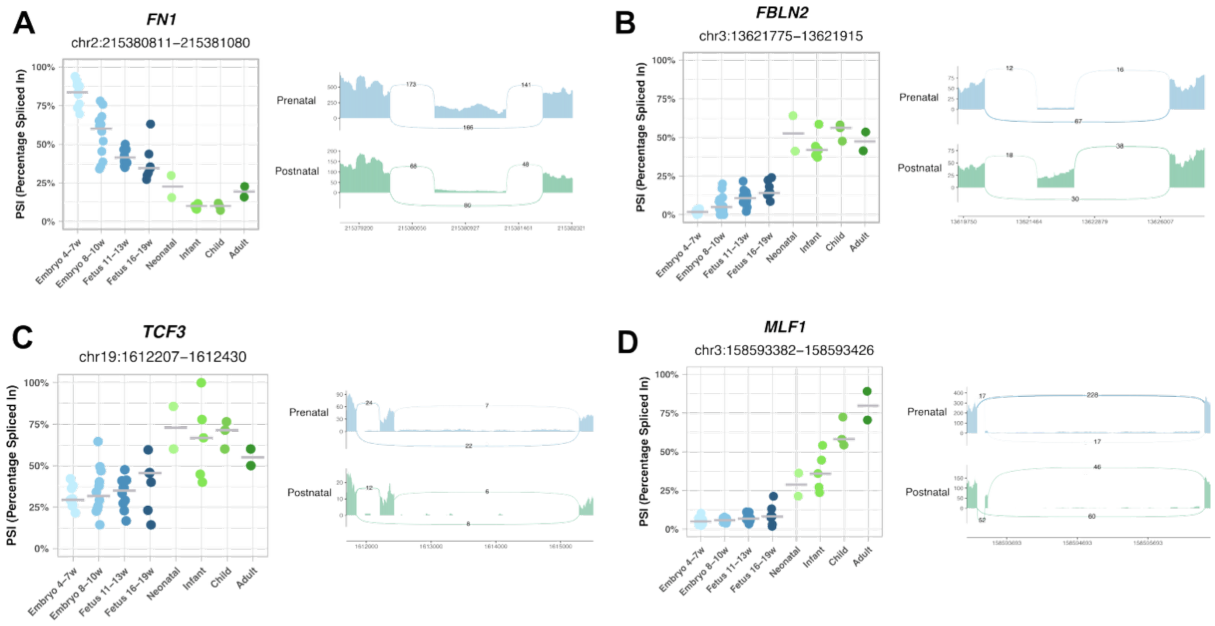

Supplementary Figure 2. Pre- to postnatal splicing transitions in the heart

A-D. Inclusion levels (PSI) of exons differentially included between prenatal and postnatal hearts, specifically (**A**) *FN1* exon 33 (*FN1*-203), (**B**) *FBLN2* exon 9 (*FBLN2*-203), (**C**) *TCF3* exon 18 (*TCF3*-203), (**D**) *MLF1* exon 3 (*MLF1*-203). PSI levels are shown for prenatal and postnatal hearts, across various developmental time points. Each event is accompanied by the corresponding sashimi plot.

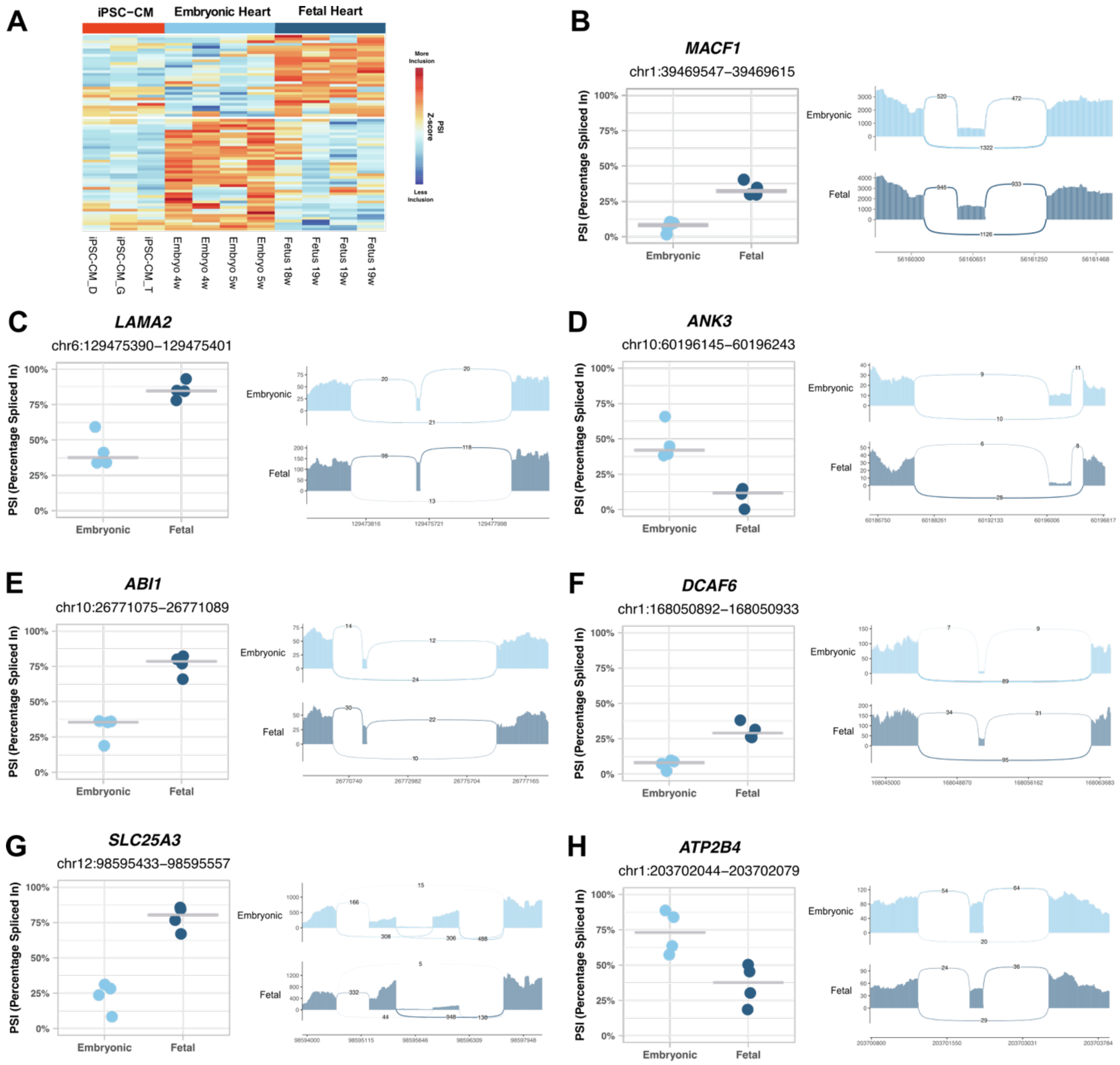

Supplementary Figure 3. Splicing regulation in the developing prenatal hearts

A. Heatmap displaying Z-score normalized Percent Spliced In (PSI) levels of all intron retention found when comparing 4-5 weeks embryonic with 18-19 weeks fetal hearts. Colors represent inclusion levels relative to the mean for each event (red indicates higher inclusion, blue indicates lower inclusion).

B-H. Inclusion levels (PSI) of exons differentially spliced between 4-5 weeks embryonic and 18-19 weeks fetal hearts, specifically (**B**) *MYO18A* exon 11 (*MYO18A*-203), (**C**) *LAMA2* exon 53 (*LAMA2*-201), (**D**) *ANK3* exon 16 (*ANK3*-201), (**E**) *ABI1* exon 4 (*ABI1*-206), (**F**) *DCAF6* exon 17 (*DCAF6*-202), (**G**) *SLC25A3* exon 3 (*SLC25A3*-202) and (**H**) *ATP2B4* exon 7 (*ATP2B4*-203). PSI levels are shown for embryonic hearts and fetal hearts. Each event in accompanied the corresponding sashimi plot.

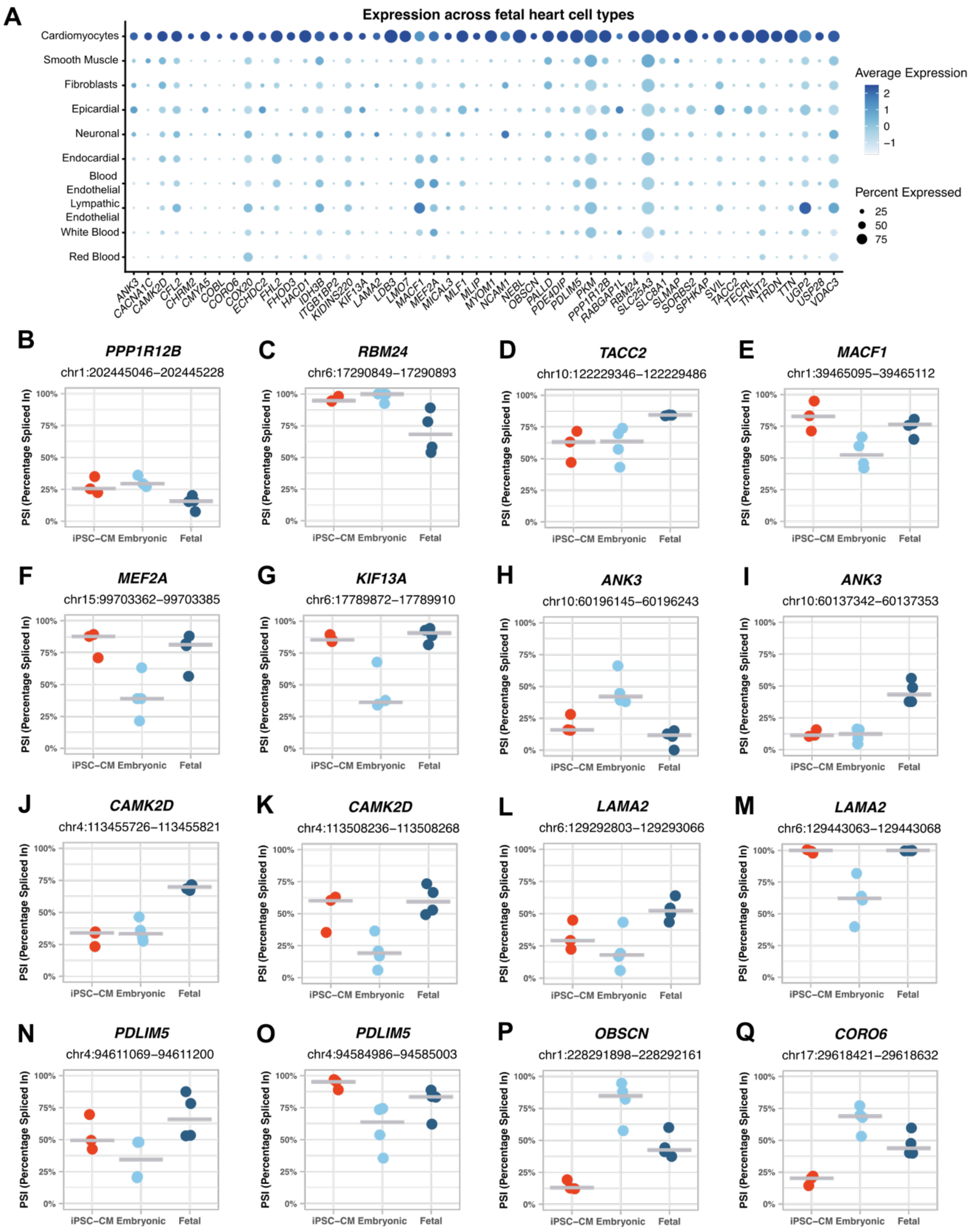

Supplementary Figure 4. Heterogeneous mirroring of prenatal heart splicing regulation in iPSC-CMs

A. Dot plot depicting the expression of cardiomyocyte-specific genes (log2FC > 0.5 compared with all other cell types) across the fetal heart single-cell atlas. Dot size represents the percentage of cells expressing each gene, and dot color indicates the scaled expression levels averaged per cell population.

B-Q. Inclusion levels (PSI) of exons differentially spliced between 4-5 weeks embryonic and 18-19 weeks fetal hearts, specifically **(B)** *PPP1R12B* exon 13 (*PPP1R12B*-204), (**C**) *RBM24* exon 4 (*RBM24*-207), **(D)** *TACC2* exon 15 (*TACC2*-210), (**E**) *MACF1* exon 95 (*MACF1*-230), (**F**) *MEF2A* exon 9 (*MEF2A*-205), (**G**) *KIF13A* exon 26 (*KIF13A*-201), (**H**) *ANK3* exon 16 (*ANK3*-201), (I) *ANK3* exon 25 (*ANK3*-209), (**J**) *CAMK2D* exon 20 (*CAMK2D*-211), (**K**) *CAMK2D* exon 14 (*CAMK2D*-237), (**L**) *LAMA2* exon 21 (*LAMA2*-206), (**M**) *LAMA2* exon 44 (*LAMA2*-201), (**N**) *PDLIM5* exon 11 (*PDLIM5*-222), (**O**) *PDLIM5* exon 6 (*PDLIM5*-207), (**P**) *OBSCN* exon 44 (*OBSCN*-228), (**Q**) *CORO6* exon 4 (*CORO6*-216). PSI levels are shown for iPSC-CMs, embryonic hearts and fetal hearts.

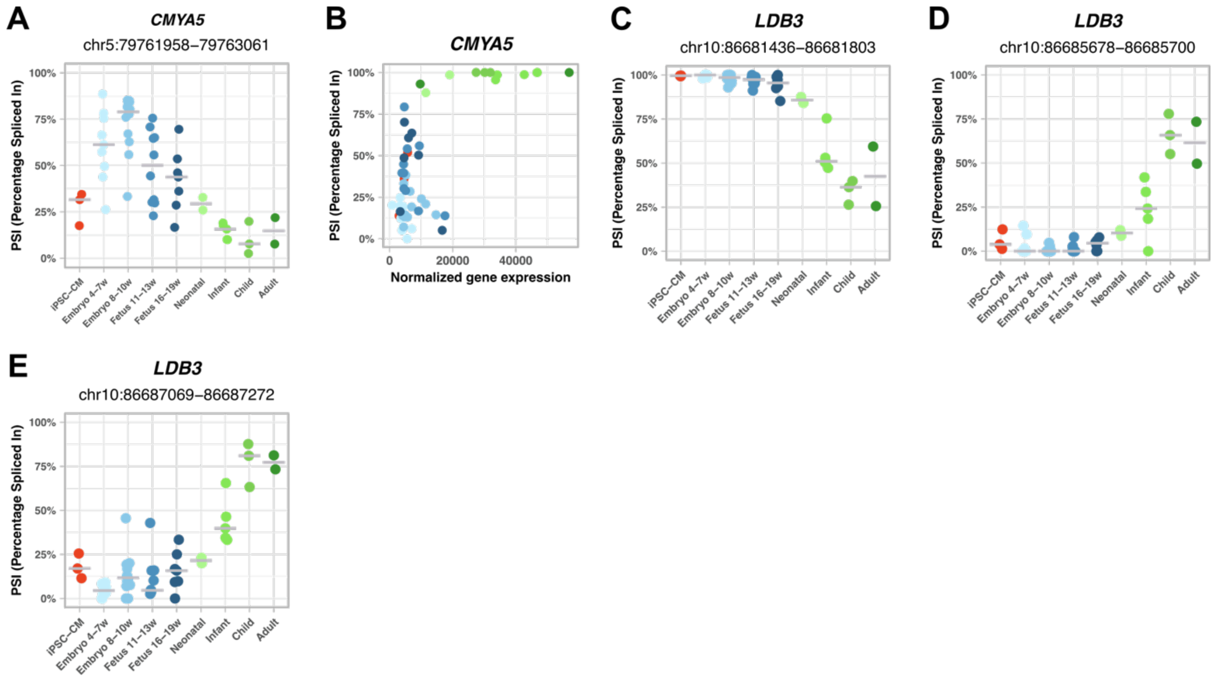

Supplementary Figure 5. Maturation-driven splicing changes in iPSC-CMs recapitulate developmental transitions in the human heart

A, C-E. Inclusion levels (PSI) of exons/introns differentially spliced between prenatal and postnatal hearts, specifically (**A**) *CMYA5* intron 8 (*CMYA5*-201), (**C**) *LDB3* exon 5 (*LDB3*-202), (**D**) *LDB3* exon 5 (*LDB3*-205) and (**E**) *LDB3* exon 6 (*LDB3*-205). PSI levels are shown for iPSC-CMs, prenatal hearts, and postnatal hearts across various developmental time points. **B.** Correlation between the Percent Spliced In of *CMYA5* exon 5, which is predicted to lead to NMD when spliced out, and *CMYA5* gene expression levels.

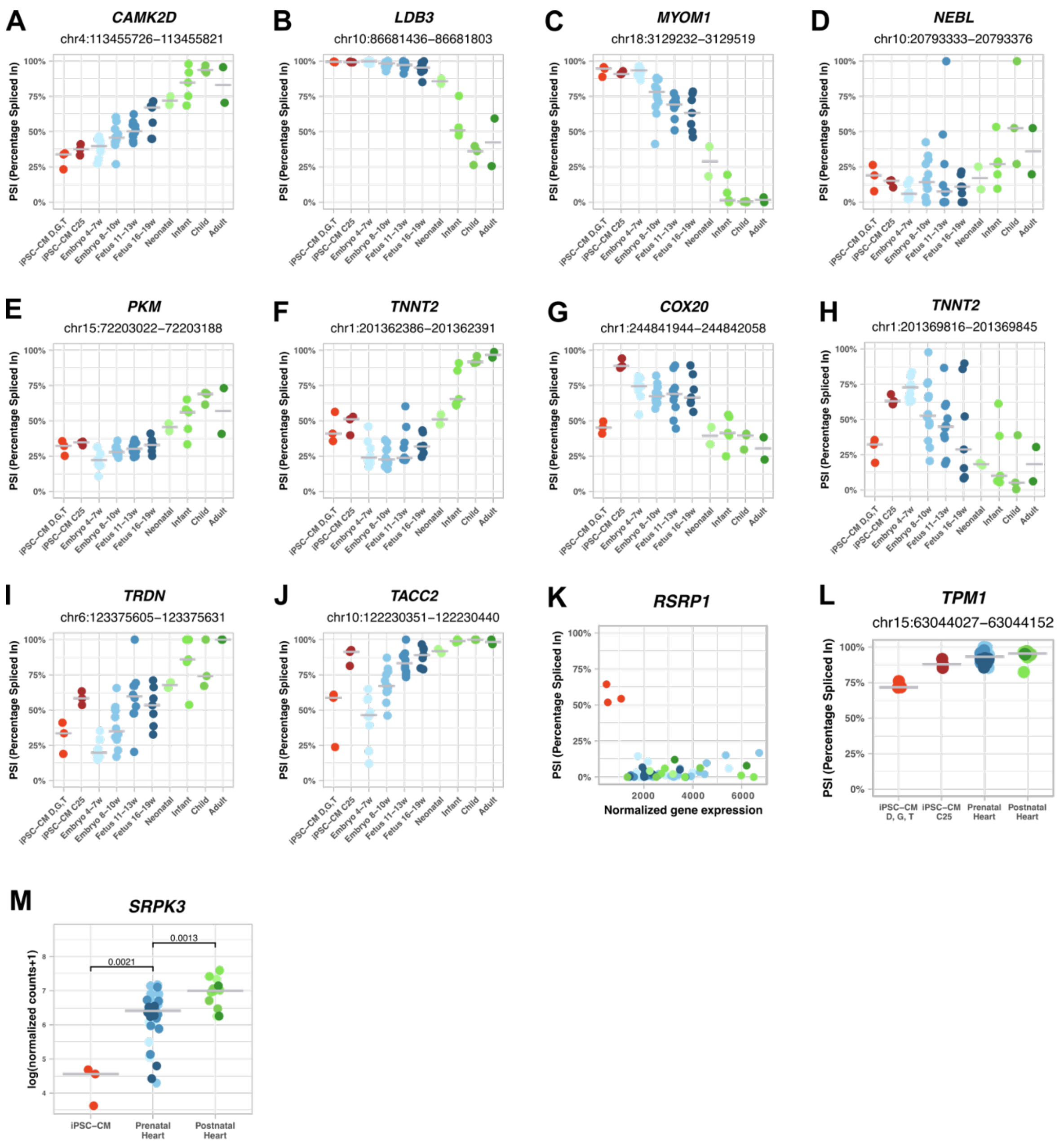

Supplementary Figure 6. Protocol-independent iPSC-CM–specific splicing events.

A-J. Inclusion levels (PSI) of exons in iPSC-CMs and both prenatal and postnatal hearts, specifically (**A**) *CAMK2D* exon 20 (*CAMK2D*-211), (**B**) *LDB3* exon 5 (*LDB3*-205), (**C**) *MYOM1* exon 18 (*MYOM1*-202), (**D**) *NEBL* exon 6 (*NEBL*-224), (**E**) *PKM* exon 9 (*PKM*-219), (**F**) *TNNT2* exon 10 (*TNNT2*-203), (**G**) *COX20* exon 2 (*COX20*-203), (**H**) *TNNT2* exon 8 (*TNNT2*-202), (**I**) *TRDN* exon 19 (*TRDN*-201), (**J**) *TACC2* exon 16 (*TACC2*-210). PSI levels are shown for iPSC-CMs purified by fluorescence-activated cell sorting with VCAM1 antibodies (iPSC-CMs D, G, T), iPSC-CMs purified by metabolic selection with lactate (iPSC-CM C25), prenatal hearts, and postnatal hearts.

K. Correlation between the Percent Spliced In of *RSRP1* exon 4, which is predicted to lead to NMD when included, and *RSRP1* gene expression levels (DESeq2 normalized counts).
L. Inclusion levels (PSI) of *TPM1* exon 2 (*TPM1*-207) across iPSC-CMs purified by fluorescence-activated cell sorting with VCAM1 antibodies (iPSC-CMs D, G, T), iPSC-CMs purified by metabolic selection with lactate (iPSC-CM C25), prenatal and postnatal hearts.
M. Normalized expression of *SRPK3* in iPSC-CMs, prenatal hearts, and postnatal hearts.

Supplementary Tables

Table S1. Sequencing metrics of single-cell and bulk RNA-sequencing from iPSC-CMs.
Table S2. Differentially spliced events detected between prenatal and postnatal hearts.
Table S3. Differentially spliced events detected between iPSC-CMs and prenatal hearts, and between iPSC-CMs and postnatal hearts.
Table S4. Selected AS events between prenatal and postnatal hearts, along with their predicted functional impact.
Table S5. Differentially spliced events detected between embryonic and fetal hearts.
Table S6. Selected AS events between embryonic and fetal hearts, along with their predicted functional impact.
Table S7. Cardiomyocyte-enriched genes (genes with log2FC > 0.5 when comparing cardiomyocytes with all other cell types in the fetal heart single-cell atlas)
Table S8. Proteomic validation of developmentally regulated splice isoforms.
Table S9. Splicing events with divergent patterns in iPSC-CMs and hearts.
Table S10. Selected iPSC-CM-specific AS events, along with their predicted functional impact.
Table S11. RBPs differentially expressed between iPSC-CMs and both prenatal and postnatal hearts.
Table S12. AS-specific primers for quantitative PCR.

Table S1. Sequencing metrics of single-cell and bulk RNA-sequencing from iPSC-CMs.

**Bulk RNA-seq**				
	**Total Read Counts**	**Processed Read Counts**	**Uniquely mapped reads**	**Uniquely mapped reads (%)**
**iPSC-D**	82715016	82022464	77197185	94.12
**iPSC-G**	91864255	91525330	85315353	93.22
**iPSC-T**	83456425	82715736	77354699	93.52

**Single-cell RNA-seq**			
**iPSC-T**		**Before Filtering**	**After Filtering**
	Number of Cells	8536	6783
	Total Number of Reads	202544724	184193016
	Mean Reads per Cell	23728	27155
	Median UMI Counts per Cell	21822	23970
	Median Genes per Cell	4626	4908
**iPSC-D**		**Before Filtering**	**After Filtering**
	Number of Cells	4283	3516
	Total Number of Reads	96006517	92823424
	Mean Reads per Cell	22405	26400
	Median UMI Counts per Cell	21312	24047
	Median Genes per Cell	5003	5373

Table S4. Selected AS events between prenatal and postnatal hearts, along with their predicted functional impact.

Functional category	Gene	Exon	PSI Prenatal Hearts	PSI Postnatal Hearts	Affected domain/ motif
Metabolism	***ACSS2***	chr20:34915218-34915256	0.43	0.87	AMP-binding domain (PF00501)
Muscle Contraction	***ACTN4***	chr19:38710257-38710342	0.59	0.83	Calponin homology (CH) domain (PF00307)
Ion Channel	***ANK2***	chr4:113378090-113378181	0.16	0.54	Disordered region
Ion Channel	***CAMK2D***	chr4:113455726-113455821	0.46	0.88	Disordered region
Sarcomeric	***CMYA5***	chr5:79747091-79747113	0.21	0.99	ORF disruption upon exon exclusion<br>GLU_RICH<br>Disordered region
Cytoskeleton	***COBL***	chr7:51187914-51187958	0.02	0.32	Cordon-bleu ubiquitin-like domain (PF09469)<br>Disordered region
ECM organization	***DCN***	chr12:91179362-91179686	0.03	0.31	-
Transcription	***DMPK***	chr19:45771350-45771396	0.95	0.74	-
ECM organization	***FBLN2***	chr3:13621775-13621915	0.08	0.46	Calcium-binding EGF domain (PF00307)
ECM/ Cell-cell communication	***FERMT2***	chr14:52861587-52861610	0.04	0.43	FERM central domain (PF00373)
ECM	***FGFR1***	chr8:38429682-38429948	0.68	0.38	Immunoglobulin (Ig) domain (PF00047)
ECM	***FN1***	chr2:215392931-215393203	0.46	0.11	Fibronectin type III domain (PF00041)<br>Disordered region
ECM	***FN1***	chr2:215380811-215381080	0.48	0.11	Fibronectin type III domain (PF00041)<br>Disordered region
Translation	***FXR1***	chr3:180971075-180971155	0.55	0.80	-
Transcription	***HMGN3***	chr6:79202063-79202155	0.31	0.68	Nucleosomal binding domain (PF01101)

					Disordered region
Mitochondrial	***IDH3B***	chr20:2658395-2658522	0.40	0.18	Isocitrate/isopropylmalate dehydrogenase domain (PF00180)
Mitochondrial	***IMMT***	chr2:86170749-86170841	0.57	0.18	PF097314 Mitofilin<br>Disordered region
Transcription	***IWS1***	chr2:127485874-127486033	0.13	0.52	Disordered region
Sarcomeric	***LDB3***	chr10:86706531-86706719	0.70	0.23	Disordered region<br>PS50099 PRO_RICH<br>PS50310 ALA_RICH
Sarcomeric	***LDB3***	chr10:86681436-86681803	0.98	0.51	-
Sarcomeric	***LDB3***	chr10:86685678-86685700	0	0.38	Disordered region
Sarcomeric	***LDB3***	chr10:86687069-86687272	0.09	0.55	Domain of unknown function (PF15936)<br>Often contains Zasp-like motif
Histone	***MACRO H2A1***	chr5:135352946-135353045	0.42	0.15	ORF disruption upon sequence exclusion<br>Macro domain (PF0166116). Binds ADP-ribose
Splicing	***MBNL1***	chr3:152446704-152446757	0.47	0.12	PS50310 Ala-Rich
Transcription	***MEF2D***	chr1:156476494-156476514	0.09	0.46	Disordered region
Mitochondrial	***MFF***	chr2:227342745-227342819	0.37	0.58	Miff domain (PF05644)<br>Disordered region
Cell cycle	***MLF1***	chr3:158593382-158593426	0.06	0.49	Mlf1IP domain (PF10248)<br>Disordered region
Sarcomeric	***MYL6***	chr12:56160626-56160670	0.36	0.82	-
Sarcomeric	***MYO1B***	chr2:191400749-191400835	0.57	0.12	PS50096 IQ
Sarcomeric	***MYO1B***	chr2:191402632-191402718	0.59	0.29	PS50096 IQ
Sarcomeric	***MYOM1***	chr18:3129232-3129519	0.75	0.01	Disordered region between FN3 domains
ECM	***NCAM1***	chr11:113242805-113242846	0.07	0.76	-
Transcription	***NCOR2***	chr12:124327547-124327633	0.83	0.41	-

Mitochondrial	***PKM***	chr15:72203022-72203188	0.28	0.57	Alpha/beta domain (PF02887), Barrel domain (PF00224)
Endosomal trafficking	***PLEKHM2***	chr1:15721329-15721388	0.72	0.98	Binding motifs of TPR domain of kinesin light chain 1 Disordered region
Mitochondrial	***SLC25A3***	chr12:98595433-98595557	0.60	0.87	Mitochondrial carrier protein PF00153
Muscle Contraction	***SLMAP***	chr3:57907884-57908006	0.38	0.14	Filament domain (PF00038) Disordered region
Mitochondrial	***STAU2***	chr8:73527691-73527804	0.29	0.63	Staufen C-terminal domain (PF16482)
Transcription	***TAF1***	chrX:71459150-71459251	0.35	0.14	-
Transcription	***TCF3***	chr19:1612207-1612430	0.34	0.63	Helix-loop-helix DNA-binding domain (PF00010) Co-resolved interactions with TAL1, LMO2, ID1 and NEUROD1
Transcription	***THOC5***	chr22:29531078-29531110	0.38	0.81	PF09766
Vesicular Transport	***TMED2***	chr12:123587611-123587631	0.30	0.56	GOLD-like domain (PF01105)
Muscle Contraction	***TNNT2***	chr1:201362386-201362391	0.25	0.89	Troponin domain (PF00992)
Muscle Contraction	***TNNT2***	chr1:201369816-201369845	0.94	0.61	-
Muscle Contraction	***TPM3***	chr1:154172029-154172104	0.71	0.37	Coiled coil containing alternating actin binding sites (PF00261)
Ion Channel	***TRDN***	chr6:123375605-123375631	0.42	0.85	LYS_RICH Disordered region
Vesicular Transport	***USO1***	chr4:75795336-75795356	0.04	0.49	Uso1/p115 like vesicle tethering protein, head region (PF04869)
Metabolism	***VDAC3***	chr8:42396678-42396680	0.57	0.83	Porin (PF01459)

Table S6. Selected AS events between embryonic and fetal hearts, along with their predicted functional impact.

Functional Category	Gene	Exon	PSI 1 month Heart	PSI 5 month Heart	Affected domain / motif
Cytoskeleton	***ABI1***	chr10:26771075-26771089	0.36	0.79	Abi-interactor HHR (PF07815)
Scaffolding	***ANK2***	chr4:113323759-113323794	0.23	0.55	ZU5 domain (PF00791, PS51145)
Scaffolding	***ANK3***	chr10:60196145-60196243	0.42	0.12	Ankyrin repeats (PF13637, PS50088)
Chromatin	***ARID1B***	chr6:157174847-157175005	0.74	0.96	Serine rich region (PS50324)<br>Highly disordered region
Ion	***ATP2B4***	chr1:203702044-203702079	0.73	0.38	E1-E2 ATPase (PF00122)<br>Highly disordered region
Transcription	***DCAF6***	chr1:168050892-168050933	0.08	0.29	Nuclear localization signal (PS50079)<br>IQ motif (PS50096)<br>Highly disordered region
Cytoskeleton	***EML1***	chr14:99892139-99892195	0.08	0.58	Hydrophobic EMAP-Like Protein (HELP) motif (PF03451)
Extracellular Matrix	***FXR1***	chr3:180971075-180971155	0.50	0.86	Nuclear localization signal (PS50079)<br>Arginine rich region (PS50323)<br>Highly disordered region
Histone	***H2AFY***	chr5:135350823-135350913	0.47	0.79	ORF disruption upon sequence inclusion<br>Macro domain (PF01661, PS51154)
Histone	***H2AFY***	chr5:135352946-135353045	0.57	0.24	ORF disruption upon sequence exclusion<br>Macro domain (PF01661, PS51154)
Mitochondrial	***IDH3B***	chr20:2658638-2658837	0.40	0.11	Isocitrate/isopropylmalate dehydrogenase domain (PF00180)
ECM	***LAMA2***	chr6:129292803-129293066	0.18	0.52	Laminin EGF domain (PF00053, PS50027)<br>Cysteine-rich region (PS50311)
ECM	***LAMA2***	chr6:129443063-129443068	0.62	1	Laminin Domain II (PF06009)<br>Highly disordered region
ECM	***LAMA2***	chr6:129475390-129475401	0.44	0.86	Laminin G domain (PF00054, PS50025)
Cytoskeleton	***MACF1***	chr1:39465095-39465112	0.52	0.76	Growth-Arrest-Specific Protein 2 (GAS2) Domain (GAS2) (PF02187, PS51460)
Splicing	***MBNL2***	chr13:97356796-97356849	0.60	0.17	Highly disordered region
Microtubules	***MDM1***	chr12:68316581-68316610	0.23	0.66	MDM1 domain (PF15501)<br>Highly disordered region
Cytoskeleton	***MYO5A***	chr15:52331699-52331836	0	0.37	ORF disruption upon sequence inclusion<br>Highly disordered region
Cytoskeleton	***PDLIM7***	chr5:177491404-177491420	0.41	0.68	ORF disruption upon sequence inclusion<br>Highly disordered region
mRNA processing	***RBM26***	chr13:79353153-79353224	0.24	0.72	Highly disordered region
Cytoskeleton	***SLAIN2***	chr4:48394576-48394653	0.27	0.66	SLAIN motif-containing family (PF15301)<br>Highly disordered region
Mitochondrial	***SLC25A3***	chr12:98595433-98595557	0.50	0.94	Mitochondrial carrier protein (PF00153, PS50920)
Mitochondrial	***SLC25A3***	chr12:98595727-98595848	0.48	0.07	ORF disruption upon sequence exclusion<br>Mitochondrial carrier protein (PF00153, PS50920)
Cell Cycle	***SYNE1***	chr6:152168113-152168281	0	0.23	Spectrin repeat (PF00435)
Muscle Contraction	***TPM3***	chr1:154172029-154172104	0.92	0.59	Tropomyosin (PF00261)
Ion Channel	***TRDN***	chr6:123269849-123269866	0.38	0.91	Lysine-rich region (PS50318)<br>Highly disordered region
Angiogenesis	***VEGFA***	chr6:43780732-43780803	0.14	0.47	VEGF heparin-binding domain (PF14554)

Table S8. Proteomic validation of developmentally regulated splice isoforms.

Gene Names	Samples	Splicing Events	Genome Coordinates	Peptides (MS/MS counts ratio)	PSI
MYOM1	iPSC-CMs (n = 3)	Exon 18 inclusion	chr18:3129232-3129519	AAIGGGVSPDVCPALSDEPGGLTASR; VSETVQEELTPPPQK N = 4 (82)	0.95
		Exon 18 skipping		- N = 0 (82)	
	Adult Heart (n = 18)	Exon 18 inclusion		AAIGGGVSPDVCPALSDEPGGLTASR; VHEASPPTFQK N = 14 (12355)	0.01
		Exon 18 skipping		AAIAPPSPPCDITCLESFR; AAIAPPSPPCDITCLESFRDSMVLGWK N = 116 (12355)	
ACTN4	iPSC-CMs (n = 3)	Exon 8 inclusion	chr19:38710257-38710342	MLDAEDIVNTARPDEK N = 2 (46)	0.51
		Exon 8 skipping		- N = 0 (46)	
	Adult Heart (n = 18)	Exon 8 inclusion		MLDAEDIVNTARPDEK N = 132 (2580)	0.83
		Exon 8 skipping		- N = 0 (2580)	
HMGN3	iPSC-CMs (n = 3)	Exon 6 inclusion	chr6:79202063-79202155	STVNVSTSR N = 2 (6)	0.31
		Exon 6 skipping		AEEAQKTESVDNEGE N = 1 (6)	
	Adult Heart (n = 18)	Exon 6 inclusion		AEEIHISR; STVNVSTSR N = 53 (53)	0.68
		Exon 6 skipping		- N = 0 (53)	
CAMK2D	iPSC-CMs (n = 3)	Exon 20 inclusion	chr4:113455726-113455821	SGSPTVPIK; ENFSGGTSLWQNI N = 3 (24)	0.34
		Exon 20 skipping		- N = 0 (24)	
	Adult Heart (n = 18)	Exon 20 inclusion		SGSPTVPIK N = 20 (948)	0.88
		Exon 20 skipping		SGSPTVPIN N = 1 (948)	

Table S10. Selected iPSC-CM-specific AS events, along with their predicted functional impact.

Functional category	Gene	Exon Coordinates	PSI iPSC-CM	PSI Prenatal Hearts	PSI Postnatal Hearts	Affected Domains/ Motifs
Kinase	***CLK1***	chr2:200860125<br>-200860215	0.38	0.95	0.97	ORF disruption upon exon exclusion<br>Protein kinase domain (PF00069, PS50011)
Kinase	***CLK4***	chr5:178617344<br>-178617434	0.17	0.96	1	ORF disruption upon exon exclusion<br>Protein kinase dmain (PF00069, PS50011)
Cell Adhesion	***ITGB1***	chr10:32907063<br>-32907143	0.22	1	1	Integrin beta cytoplasmic domain (PF08725)<br>Phosphotyrosine binding domain<br>(ELME000122)
Methylation	***METTL3***	chr14:21499372<br>-21499491	0.47	0.82	0.83	ORF disruption upon intron inclusion<br>Disrupts MT-A70 domain (PF05063)
mRNA decay	***PATL1***	chr11:59655956<br>-59656045	0.79	1	1	Topoisomerase II-associated protein PAT1 (PF09770)<br>Proline-rich region (PS50099)<br>Highly disordered region
Cell cycle	***PDE4DIP***	chr1:149024445<br>-149024684	0.13	0	0	Highly disordered region
Splicing	***RSRP1***	chr1:25244454-<br>25244498	0.54	0.02	0.02	ORF disruption upon exon inclusion<br>Highly disordered region
Splicing	***SNRPE***	chr1:203862182<br>-203862222	0.56	0.92	1	LSM domain (PF01423)
Contraction	***TPM1***	chr15:63044027<br>-63044152	0.72	0.93	0.95	Tropomyosin domain<br>(PF00261)
NMD	***UPF3A***	chr13:11428630<br>2-114286400	0.61	1	1	Smg-4/UPF3 family (PF03467)<br>Glutamic acid-rich region (PS50313);<br>Disordered region

Table S11. RBPs differentially expressed between iPSC-CMs and both prenatal and postnatal hearts.

Gene ID	Gene Name	log2FoldChange	adjusted p-value	Direction
ENSG00000060339	*CCAR1*	1.91620618021671	0.00780583690621775	Upregulated in Postnatal Hearts
ENSG00000060339	*CCAR1*	2.04348449225204	0.00156240494279396	Upregulated in Prenatal Hearts
ENSG00000013441	*CLK1*	2.93955240561227	0.000415084972412897	Upregulated in Postnatal Hearts
ENSG00000013441	*CLK1*	2.88310912365033	0.000405509925248229	Upregulated in Prenatal Hearts
ENSG00000196361	*ELAVL3*	4.72675144411945	0.000955901273861579	Upregulated in Postnatal Hearts
ENSG00000196361	*ELAVL3*	4.67173240084773	0.00105372978384178	Upregulated in Prenatal Hearts
ENSG00000162374	*ELAVL4*	6.18871886393131	0.0019636194517663	Upregulated in Postnatal Hearts
ENSG00000162374	*ELAVL4*	10.1767754651497	1.49311611725135e-06	Upregulated in Prenatal Hearts
ENSG00000104967	*NOVA2*	4.07963840531238	4.6476120519874e-10	Upregulated in Postnatal Hearts
ENSG00000104967	*NOVA2*	3.60589089468299	3.05475256771898e-08	Upregulated in Prenatal Hearts
ENSG00000184343	*SRPK3*	3.69125357208732	7.64817269120447e-07	Upregulated in Postnatal Hearts
ENSG00000184343	*SRPK3*	2.86984847300263	0.000390130921686436	Upregulated in Prenatal Hearts
ENSG00000169554	*ZEB2*	2.08712447344409	0.00280618656988859	Upregulated in Postnatal Hearts
ENSG00000169554	*ZEB2*	2.24249942801933	0.000384214664629045	Upregulated in Prenatal Hearts

Table S12. AS-specific primers for quantitative PCR.

Primer	Sequence	Type	Amplicon Size
**Maturation-driven splicing events**			
TNNT2 Ex5 Fw	GAGGACTGGAGAGAGGACGA	target AS event	122 bp
TNNT2 Ex7 Rv	GCCTCCTTTGCTTCCTCTTCT		
TNNT2 Ex14 Fw	AGCGGAAAAGTGGGAAGAGG	constitutive exon	103 bp
TNNT2 Ex14 Rv	AGCTGATCTTCATTCAGGTGGT		
CMYA5 Ex4 Fw	TCTGCAGAGCATGGACACTG	target AS event	101 bp
CMYA5 Ex4 Rv	TGATTTCCTCAAACGAAGTCAGG		
CMYA5 Ex8 Fw	ACAGTGAAAGAAAGCTACTGCA	constitutive exon	107 bp
CMYA5 Ex8 Rv	CTTTCACTGGGCAGGCTACA		
LDB3 Ex8 Fw	CTGCTTCAAGTCCTGCCGA	target AS event	101 bp
LDB3 Ex8 Rv	CTCACTGTAGCTGGTGTGGG		
LDB3 Ex6 Fw	AAGGACCTTGCCGTAGACAG	constitutive exon	114 bp
LDB3 Ex6 Rv	AGACTGCAGGTTGGAGGAAC		
TRDN Ex19 Fw	GAACACTCAGTTCCAAGTGA	target AS event	115 bp
TRDN Ex20 Rv	CTTTTTTAATTGAAACCGCA		
TRDN Ex2 Fw	TGGATCTGTGCCCAAATCCC	constitutive exon	95 bp
TRDN Ex2 Rv	TCAGGGCAATGACCAGAAGC		
CAMK2D Ex17 Fw	TGCCAAAGACAATGCAGTCAG	target AS event	201 bp
CAMK2D Ex18 Rv	AGACCCATATGTGAATGGTTTTCA		
CAMK2D Ex10 Fw	GGACACGGTGACTCCTGAAG	constitutive exon	102 bp
CAMK2D Ex10 Rv	ATCCATGGGTGCTTCAGTGC		
**iPSC-CM splicing events**			
CLK1 Ex4Fw	GGATGATGAGGAGGGTCACC	target AS event	117 bp
CLK1 Ex5Rv	TGATCGATGCACTCCACAACT		
CLK1 Ex9Fw	CGTGATGAACGCACCTTAATAA	constitutive exon	116 bp
CLK1 Ex9Rv	TGATCGATGCACTCCACAACT		
CLK4 Ex4Fw	GGATGATGAGGAGGGTCACC	target AS event	110 bp
CLK4 Ex5Rv	TGCACTCTACAACTTTGCCA		
CLK4 Ex2Fw	GCGGCATTCCAAAAGAACTCA	constitutive exon	123 bp
CLK4 Ex2Rv	TGCCTGTTCTCTTGTGTGCT		
SNRPE Ex1Fw	GTGGCCAGGGTCAGAAAGTG	target AS event	103 bp
SNRPE Ex2Rv	TCTGAAGATGAGGTTCTGCAC		
SNRPE Ex5Fw	ACTCTGCTACAAAGTGTCTCCA	constitutive exon	146 bp
SNRPE Ex5Rv	GCCATCTTGTAGTAACACGAGG		
METTL3 Int8Fw	GGGCCCAATTCAATAGGTGGA	target AS event	143 bp
METTL3 Ex9Rv	CTGGTTGAAGCCTTGGGGAT		
METTL3 Ex2Fw	CTACGGAATCCAGAGGCAGC	constitutive exon	216 bp
METTL3 Ex2Rv	CGTGGAGATGGCAAGACAGA		

Supplementary Experimental Procedures

Immunofluorescence image acquisition and analysis

Fluorescence images were acquired with Zeiss LSM 710 Confocal Laser Point-Scanning Microscope. Quantitative image analysis was performed using ImageJ (https://imagej.nih.gov/) standard plugins. For sarcomere length measurements, statistical analysis was performed using Brown-Forsythe and Welch ANOVA tests to account for unequal variances, followed by Games-Howell's multiple comparisons test.

Quantitative RT-PCR (qRT-PCR)

Total RNA was extracted using NZYol (NZYTech®) and digested with DNase I (Roche®). cDNA synthesis was performed the SuperScript IV Reverse Transcriptase (Invitrogen^{TM}) with oligodT primers. qRT-PCR was carried out using the Universal SYBR Green Supermix (Bio-Rad) and specific primers designed for the selected splicing events (listed in **Table S12**). Each PCR reactions was run in triplicate, using the ViiA™7 RT-PCR Systems (Applied BioSystems). Quantification was performed using the 2–ΔCt method. The level of the target exon was normalized to the level of a constitutive exon from the same gene (i.e., an exon that is consistently included in all splicing isoforms transcribed from the gene). For statistical analysis we used Brown-Forsythe and Welch ANOVA tests to account for unequal variances, followed by Games-Howell's multiple comparisons test.

Single-cell RNA sequencing

At day 30, differentiated cells were dissociated using 0.25% Trypsin-EDTA (Gibco) for 5 min at 37ºC. After stopping the reaction, dissociated cells were resuspended in PBS/0.1% bovine serum albumin (BSA; Life Technologies), filtered through a 100 µm pore filter and stored in ice. Cell viability was assessed by manual counting using Trypan Blue. Viable cell suspensions (1x106 cells/mL) were partitioned into Gel Beads-in-emulsion (GEMs), in order to capture approximately 10000 cells in single cell droplets, using the Chromium Single Cell 3′ GEM, Library & Gel Bead Kit (version 3, 10X Genomics, PN-1000092) and Chromium Single Cell B Chip Kit (10X Genomics, PN-1000074). Barcoded cDNA was extracted from the GEMs by

Post-GEM RT-cleanup, PCR amplified and subjected to quality control and quantification. Single Cell 3′ Gene Expression libraries from single cells was then performed using Chromium i7 Multiplex Kit (PN-120262). All the steps were performed according to the manufacturer's protocol. Libraries were sequenced using an Illumina NextSeq 500 Instrument with a 300 cycles kit. The raw FASTQ files have been uploaded to ArrayExpress and can be accessed using the accession number E-MTAB-13850.

Single-cell RNA sequencing analysis

Raw sequencing reads from FASTQ files were pseudo-aligned to the transcriptome using Kallisto BUStools (Melsted et al., 2021). The resulting count matrix was imported into R and converted into a Seurat object using Seurat v.5.1.0 (Hao et al., 2021). To retain high-quality cell-containing droplets, emptyDrops from DropletUtils v1.24.0 (participants in the 1st Human Cell Atlas Jamboree et al., 2019) was used with a false discovery rate (FDR) of ≤ 0.01. Cells were filtered according to their number of expressed genes (≥ 500), number of UMIs (≥ 1000), log10GenesPerUMI (> 0.8) and ribosomal ratio (> 0.05). The threshold for the expression of mitochondrial genes was set to 40% given the high energy needs of cardiomyocytes (Mercer et al., 2011). We further kept only those genes expressed in more than 10 cells in each sample. Counts were normalized, adjusted for variance, and the 1000 most highly variable features were identified using the SCTransform function, while regressing out sequencing depth effects. Principal component analysis was performed on these features and the five principal components explaining the most variance were used for UMAP dimensionality reduction and to build a SNN graph. Known cardiomyocyte markers were used to select an appropriate resolution that allows to capture this cell population. Clustering was done using FindClusters function, with the Louvain algorithm with multilevel refinement, and resolution set to 0.01 for iPSC-CM_T and 0.05 for iPSC-CM_D. Doublet detection and removal were performed using DoubletFinder v.2.0.4 with the first 5 principal components (PCs) and pK = 0.07 for iPSC-CM_T and pK = 0.05 for iPSC-CM_D. Cluster markers were identified using Wilcoxon's rank sum test implemented in FindAllMarkers function. We considered only positive markers, as well as genes detected in at least 25% of the cells of each population, and with a minimum log2FC of 0.5. Markers with an adjusted p-value ≤ 0.01 were ranked by $\log_2$FC and used for gene set enrichment analysis (GSEA) with gseGO function of clusterProfiler 4.12.6 (Wu et al., 2021) and org.Hs.eg.db v.3.16.0 (Carlson, 2017). For automated cell type classification, label transfer was performed using Seurat's FindTransferAnchors and TransferData functions, using

the first 25 PCs of Farah *et al.* dataset (Farah et al., 2024), using the processed Seurat object publicly available. Only cells assignments with a prediction score higher than 0.6 were kept.

Bulk RNA-sequencing

RNA was extracted using NZYol (NZYTech®) and digested with DNase I (Roche®). At least 1µg of total RNA was obtained from each culture of iPSC-CMs at day 30 of differentiation. After purification with poly-T oligo-attached magnetic beads, libraries were prepared using the NEBNext® Ultra™ Directional RNA Library Prep Kit, and sequenced on the Illumina platform Novaseq6000. The raw FASTQ files have been uploaded to ArrayExpress and can be accessed using the accession number E-MTAB-13757 (Cardoso-Moreira et al., 2019). Additionally, we used RNA sequencing data from 50 healthy human hearts - 38 prenatal and 12 postnatal, generated in a previous study. Raw data (FASTQ files) was downloaded from ArrayExpress (https://www.ebi.ac.uk/arrayexpress/), with the accession number E-MTAB-6814 39.

Data preprocessing, genome alignment and transcript quantification

Quality control of the sequenced reads was performed using *FastQC v0.11.5* (Andrews, 2010). Adaptors, low-quality bases (Phred quality <20) and reads shorter than 75bp were filtered out using *Trimgalore v0.6.4* (Krueger et al., 2021). Filtered reads were pseudo-aligned to the human transcriptome and bootstrapped transcript abundance was estimated with *Salmon v1.10.2* (Patro et al., 2017), with default parameters, 100 resamplings, fragment length distribution set to a mean of 100 bp and standard deviation of 10 bp, and correction for GC and sequencing bias. Trimmed reads were also mapped to the human reference genome (GRCh38/hg38) using the splice-aware aligner *STAR v2.7.10b* (Dobin et al., 2013). Chimeric alignments were detected (--chimSegmentMin 20), splice junctions were optimized with --sjdbOverhang 99, and two-pass mapping (--twopassMode Basic) was used to improve splice junction detection. Only uniquely mapped reads were kept using *samtools v.1.7* (Li et al., 2009). GENCODE v45 human primary gene annotation was used as reference (Frankish et al., 2019). **Table S1** summarizes the main biological and technical characteristics of all the samples.

Gene expression analysis

Transcriptomic analysis was performed using R *v4.4.1* software (R Core Team, 2021). Prior to further analysis, data was filtered so that only genes with more than 10 read counts in at least

three samples were kept. Transcript read counts were converted into a gene-level count matrix using *tximport v1.32.0* (Soneson et al., 2016). To filter out lowly expressed genes that could possibly result from transcriptional noise, only genes with transcripts per million (TPM) > 1 were considered. This threshold was determined based on the read counts distribution observed with a log10(TPM) histogram. Pearson pairwise correlation coefficients (r) were computed on rlog-transformed counts to assess the similarity between samples. The correlation matrix was visualized using *corrplot v0.*94 (Wei, Taiyun and Simko, Viktor). Venn diagrams illustrating the overlap of expressed genes (TPM > 1) between different iPSC-CM lines were generated using *VennDiagram v1.7.3* (Chen and Boutros, 2011). To prevent highly expressed genes from dominating the analysis, counts were transformed using varianceStabilizingTransformation (VST) from *DESeq2 v1.44.0* (Love et al., 2014). Principal component analysis (PCA) was performed on transformed read counts to discern the main sources of variation in the dataset using *ggplot2 v3.5.1* (Wickham, 2016). Regular hierarchical clustering dendrograms were drawn using the R base function hclust with average linkage clustering and 1-pairwise Spearman correlation coefficients as the distance metric. The *pvclust v2.2-0* (Suzuki and Shimodaira, 2006) package was used to assess the uncertainty of the clustering and to compute p-values for each cluster based on 1000 bootstrap resamplings.

Analysis of Metabolic Pathways

To evaluate pathway-specific changes in metabolism, we performed single-sample Gene Set Enrichment Analysis (ssGSEA), which enables the estimation of gene set enrichment scores across

individual samples. Curated hallmark gene sets for the Glycolysis (MSigDB ID: M5937) and Oxidative Phosphorylation (MSigDB ID: M5936) pathways were obtained from the Molecular Signatures Database (MSigDB) using the *msigdbr* package *v.24.1.0* (Igor Dolgalev, 2025). ssGSEA was applied to the VST expression matrix to quantify pathway activity for each sample using the gsva() function (Hänzelmann et al., 2013). Enrichment scores for the Glycolysis and OXPHOS pathways were visualized using violin plots to illustrate their distribution across iPSC-CMs, prenatal and postnatal hearts. To complement the pathway-level analysis, we conducted a targeted gene expression analysis focusing on key enzymes and regulators involved in cardiac metabolic processes. This allowed for finer resolution of developmental expression trends across glycolysis, the TCA cycle, oxidative phosphorylation (OXPHOS), and fatty acid oxidation (FAO). Representative genes were manually curated from each pathway based on their central metabolic roles and known developmental relevance in

cardiac tissues. A heatmap was generated using the *pheatmap* package to visualize standardized (row-scaled) VST values of the selected genes across all developmental stages.

Deconvolution of bulk RNA-seq

Cell-type proportions in heart tissue and iPSC-CM bulk RNA-seq samples were estimated using *CIBERSORTx* (Newman et al., 2019). Single-cell RNA-seq data from prenatal hearts (Farah et al., 2024) was used as reference and converted into a signature matrix using 50 replicates per cell type and retaining only genes with an average expression > 0.5 across cells. To estimate cell-type composition, *CIBERSORTx* Fractions was applied with S-mode batch correction to account for technical variability, and statistical significance was assessed with 1000 permutations for p-value calculation. A heatmap was generated to visualize cell-type proportions where atrial, ventricular, and nodal-like cardiomyocytes were merged into a single category of cardiomyocytes; red blood and white blood cells into hematopoietic; BEC, LEC and endocardial cells into endothelial cells; and fibroblasts, smooth muscle cells and epicardial into mesenchymal cells.

Expression of genes per cell-type

To explore the expression of selected genes across cardiac cell types, we used the processed Seurat object provided by the authors of Farah et al. (2024), which includes fetal human heart single-cell RNA-seq data spanning developmental stages from 9 to 15 post-conceptional weeks (Farah et al., 2024). Cardiomyocyte subtypes - atrial, ventricular, and nodal-like - were grouped under a single cardiomyocyte category, to facilitate comparisons across major cardiac lineages. A dot plot was generated to summarize expression levels across cell types for selected genes.

Splicing analysis

Alternative splicing (AS) events were identified and quantified using *rMATS v4.1.2*, *MAJIQ v2.5.1,* and *vast-tools v2.5.1* (Shen et al., 2014; Tapial et al., 2017; Vaquero-Garcia et al., 2023), which extract reads mapping to splice junctions from BAM files and calculate Percent Spliced In (PSI) values. For *rMATS*, default parameters were used (cstat 0.0001, anchorLength 3) with the -novelSS option enabled to detect unannotated splice sites. Results were filtered to retain events with ≥10 reads supporting either inclusion or skipping in at least half of the samples across at least two groups. For *MAJIQ*, a splice graph was constructed, including all binary splicing events detected in >50% of the samples per group, with a minimum of 5 supporting reads for known junctions and 7 reads for *de novo* junctions. For *vast-tools*, PSI values were

computed, including intron retention events (--p_IR). Events were retained if they had sufficient read coverage in 50% of both prenatal and postnatal groups (--min_Fr 0.5), a minimum standard deviation of 5 (min_SD 5), and met the --noVLOW criterion to exclude low-coverage events. All splicing events were further filtered to retain only those with ≤1/3 missing values in prenatal and postnatal heart samples and no missing values in iPSC-CM samples.

To assess splicing similarity between samples, Spearman pairwise correlation coefficients (ρ) were computed on logit-transformed PSI values. To determine a TPM threshold for reliable PSI estimation, splicing events were binned based on the TPM of the parent gene, and the mean PSI variance of each bin was plotted. A loess smooth curve was fitted to identify the stabilization point, determining TPM > 10 as optimal for robust splicing estimation. All splicing events detected by rMATS in genes with TPM > 10 were considered, except for constitutive events where all samples exhibited a PSI of 0 or 1. The correlation matrix was generated and visualized using *corrplot v0.94* (Wei, Taiyun and Simko, Viktor). PCA was performed using PSI values from the 500 most variable splicing events and visualized using *ggplot2 v3.5.1* (Wickham, 2016).

Significant splicing changes between groups were defined as those events with deltaPSI > +/- 0.2 and < 1% FDR for *rMATS*. With *MAJIQ* heterogen and modulize, we considered as significant binary events with a deltaPSI > +/- 0.2 and a probability of changing > 0.9. *Vast-tools* was also used to detect differentially spliced events with > 90% probability that there is a change of at least 20%.

The heatmap to visualize the differentially spliced events was generated using *pheatmap v1.0.12* (Kolde R, 2019) and *RColorBrewer v.1.1* (Neuwirth E, 2014). For this, each sample's deviation from the mean of each gene was calculated as row Z-scores, and clustering was performed using complete linkage clustering with Spearman correlation as the distance metric. Visualization of specific alternative splicing events was performed with *ggashimi v1.1.5* using the respective surrounding coordinates and the median number of supporting reads for each group (Garrido-Martín et al., 2018). The potential functional impact of the alternative splicing events was investigated using *VastDB*, by matching the event coordinates with those present in the database (Tapial et al., 2017). Unless specified otherwise, all other plots were generated using the *ggplot2 v3.5.1* package (Wickham, 2016). When multiple transcript isoforms corresponded to a specific spliced exon/intron, we reported the isoform with the highest confidence (i.e., MANE Select transcript, ENSEMBL golden isoform, or similar high-

confidence annotations), based on the GENCODE v45 human primary transcript annotation used throughout the analysis (Frankish et al., 2019).

NEASE Analysis

NEASE (Network-based Enrichment Analysis for Splicing Events) *v1.2.2* was used to infer the functional impact of the identified AS events at the protein interaction network level (Louadi et al., 2021). Exons predicted to disrupt the open reading frame (ORF) were excluded from the analysis. First, we detected protein features potentially impacted by splicing, including protein domains, linear motifs and interacting residues. Then, pathways affected by interactions mediated by the spliced features were identified. One-sided hypergeometric test was performed to identify enriched pathways, applying a p-value cutoff of 0.05. Pathways were ranked based on the NEASE score.

Sample preparation for MS analysis

Cardiomyocytes differentiated from iPSC lines D, T and C were collected at day 30 through centrifugation and the resulting pellet was stored at −80 °C. Cell pellets were dissolved in lysis buffer containing 5% sodium dodecyl sulfate (SDS) and 50 mM triethylammonium bicarbonate (TEAB), pH 8.5, and each sample was transferred to a PIXUL™ 96-well plate (Active Motif). Samples were sonicated with a PIXUL™ Multisample sonicator (Active Motif) for 5 minutes with default settings. After centrifugation for 15 min at 2204 x g at room temperature (RT), the lysates were transferred to a new plate and the protein concentration was measured by bicinchoninic acid (BCA) assay (Thermo Scientific). From each sample 50 µg of protein was isolated and volumes were adjusted to 100 µl with the lysis buffer to continue the protocol. Proteins were reduced by addition of 15 mM dithiothreitol and incubation for 30 minutes at 55˚C and then alkylated by addition of 30 mM iodoacetamide and incubation for 15 minutes at RT in the dark. Phosphoric acid was added to a final concentration of 1.2% and subsequently samples were diluted 7-fold with binding buffer containing 90% methanol in 100 mM TEAB, pH 7.55. The samples were loaded on a 96-well S-Trap™ plate (Protifi) in parts of 400 µl, placed on top of a deepwell plate, and centrifuged for 2 min at 1,500 x g at RT. After protein binding, the S-trap™ plate was washed three times by adding 200 µl binding buffer and centrifugation for 2 min at 1,500 x g at RT. A new deepwell receiver plate was placed below the 96-well S-Trap™ plate and 125 µl 50 mM TEAB containing 1 µg trypsin (1/50, w/w) was added for digestion overnight at 37°C. Using centrifugation for 2 min at 1,500 x g, peptides

were eluted in three times, first with 80 µl 50 mM TEAB, then with 80 µl 0.2% formic acid (FA) in water and finally with 80 µl 0.2% FA in water/acetonitrile (ACN) (50/50, v/v). Eluted peptides were dried completely by vacuum centrifugation.

TMTpro™ 16-plex labels (0.5 mg, lot #WF322713, Thermo Fisher Scientific) were equilibrated to RT immediately before use and dissolved in 20 µl anhydrous acetonitrile (ACN). The dried peptides were re-suspended in 90 µl 100 mM TEAB (pH 8.5), peptide concentration was determined on a Lunatic spectrophotometer (Unchained Labs) (Maia et al., 2020) and peptide amount was adjusted to 35 µg for each sample. Peptides were labeled for 1 hour at RT using 0.25 mg of TMTPro™ label (127N – C; 128C – T; 133N – D). The reaction was quenched for 15 min at RT by addition of 4.2 µl 5% hydroxylamine. The labeled samples were combined, 100 µg labeled peptides was isolated, dried by vacuum centrifugation, re-dissolved in 100 µl loading solvent A (0.1% TFA in water/ACN (98:2, v/v)), pH was adjusted with 1 µl of 100% TFA and the sample was desalted on a reversed phase (RP) C18 OMIX tip (Agilent). The tip was first washed 3 times with 100 µl pre-wash buffer (0.1% TFA in water/ ACN (20:80, v/v)) and pre-equilibrated 5 times with 100 µl of wash buffer (0.1% TFA in water) before the sample was loaded on the tip. After peptide binding, the tip was washed 3 times with 100 µl of wash buffer and peptides were eluted twice with 100 µl elution buffer (0.1% TFA in water/ACN (40:60, v/v)). The combined elutions were dried in a vacuum concentrator.

Vacuum dried peptides were re-dissolved in 100 µl loading solvent A and 95 µl (+/- 100 µg) was injected for fractionation by RP-HPLC (Agilent series 1200) connected to a Probot fractionator (LC Packings). Peptides were first loaded in loading solvent A on a 4 cm pre-column (made in-house, 250 µm internal diameter (ID), 5 µm C18 beads, Dr. Maisch) for 10 min at 25 µl/min and then separated on a 15 cm analytical column (made in-house, 250 µm ID, 3 µm C18 beads, Dr Maisch). Elution was done using a linear gradient from 100% RP-HPLC solvent A (10 mM ammonium acetate (pH 5.5) in water/ACN (98:2, v/v)) to 100% RP-HPLC solvent B (70% ACN, 10 mM ammonium acetate (pH 5.5)) in 100 min at a constant flow rate of 3 µL/min. Fractions were collected every minute between 20 and 92 minutes and pooled every 24 minutes to generate a total of 24 samples for LC-MS/MS analysis. All 24 fractions were dried under vacuum in HPLC inserts and stored at −20°C until further use.

Liquid chromatography-MS/MS analysis

Purified peptides were re-dissolved in 20 µl solvent A and 15 µl of each fraction was injected for LC-MS/MS analysis on an Ultimate 3000 RSLCnano system in-line connected to an

Orbitrap Fusion Lumos mass spectrometer equipped with a pneu-Nimbus dual ion source (Phoenix S&T). Trapping was performed at 20 µl/min for 2 min in solvent A on a 5 mm PepMap™ Neo trapping column (300 µm ID, 5 µm beads, C18, Thermo) and the sample was loaded on a 110 cm prototype µPAC column (Thermo Scientific™) with C18-endcapped functionality mounted in the Ultimate 3000's column oven at 50°C. For proper ionization, a fused silica PicoTip emitter (10 µm inner diameter) (New Objective) was connected to the µPAC™ outlet union and a grounded connection was provided to this union. Peptides were eluted by a non-linear increase from 2 to 26.4% MS solvent B (0.1% FA in water/ACN (2:8, v/v)) over 45 minutes, then to 44% in 55 minutes, first at a flow rate of 600 nl/min, then at 300 nl/min, followed by a 5-minutes wash at 60 minutes reaching 56% MS solvent B and re-equilibration with MS solvent A (0.1% FA in water).

The mass spectrometer was operated in data-dependent mode (TMT pro SPS on Lumos) with a top speed of three seconds. Full-scan MS spectra (375-1500 m/z) were acquired at a resolution of 120,000 in the Orbitrap analyzer after accumulation to a target AGC value of 400,000 with a maximum injection time of 50 ms. The precursor ions were filtered for charge states (2-7 required), dynamic exclusion (60 s; +/- 10 ppm window) and intensity (minimal intensity of 5E4). The precursor ions were selected in the quadrupole with an isolation window of 0.7 Da and accumulated to an AGC target of 1E4 or a maximum injection time of 50 ms and activated using CID fragmentation (35% NCE). The fragments were analyzed in the Ion Trap Analyzer at turbo scan rate. The 10 most intense MS2 fragments were selected in the quadrupole using MS3 multi-notch isolation windows of 2 m/z. An orbitrap resolution of 60,000 was used with an AGC target of 1E5 or a maximum injection time of 118 ms and activated using HCD fragmentation (65% NCE). The polydimethylcyclosiloxane background ion at 445.120028 Da was used for internal calibration (lock mass). QCloud was used to control instrument longitudinal performance (Chiva et al., 2018).

MS data analysis

LC-MS/MS data from the iPSC-CMs generated in this study (PRIDE accession: PXD000000) and from the publicly available dataset PXD006675 (Doll et al., 2017), which includes adult heart samples (ventricle, auricle, and septum), were analyzed using MaxQuant software (version 2.6.4.0). Searches were performed using default parameters, including a false discovery rate (FDR) of 1% at both the peptide and protein levels. Correction factors were used and extracted from TMT correction factor certificate from manufacturing lot #WF322713.

Protein identification was carried out using FASTA reference files from the UniProt human proteome (UP000005640_9606 and UP000005640_9606_additional; release 2025_04). Enzyme specificity was set to cleavage C-terminal to arginine and lysine residues, including cleavage at Arg/Lys–Pro bonds, allowing up to two missed cleavages. Carbamidomethylation of cysteine was set as a fixed modification, while methionine oxidation and N-terminal acetylation were included as variable modifications. The "match between runs" feature was enabled to enhance peptide identification across LC-MS/MS runs. Peptide identification for selected genes was extracted from the “peptides.txt” output file.

Supplementary References

Andrews S (2010). FastQC: A Quality Control Tool for High Throughput Sequence Data.

Cardoso-Moreira, M., Halbert, J., Valloton, D., Velten, B., Chen, C., Shao, Y., Liechti, A., Ascenção, K., Rummel, C., Ovchinnikova, S., et al. (2019). Gene expression across mammalian organ development. Nature *571*, 505–509. https://doi.org/10.1038/s41586-019-1338-5.

Carlson, M. (2017). org.Hs.eg.db (Bioconductor).

Chen, H., and Boutros, P.C. (2011). VennDiagram: a package for the generation of highly-customizable Venn and Euler diagrams in R. BMC Bioinformatics *12*, 35. https://doi.org/10.1186/1471-2105-12-35.

Chiva, C., Olivella, R., Borràs, E., Espadas, G., Pastor, O., Solé, A., and Sabidó, E. (2018). QCloud: A cloud-based quality control system for mass spectrometry-based proteomics laboratories. PLoS ONE *13*, e0189209. https://doi.org/10.1371/journal.pone.0189209.

Dobin, A., Davis, C.A., Schlesinger, F., Drenkow, J., Zaleski, C., Jha, S., Batut, P., Chaisson, M., and Gingeras, T.R. (2013). STAR: ultrafast universal RNA-seq aligner. Bioinformatics *29*, 15–21. https://doi.org/10.1093/bioinformatics/bts635.

Doll, S., Dreßen, M., Geyer, P.E., Itzhak, D.N., Braun, C., Doppler, S.A., Meier, F., Deutsch, M.-A., Lahm, H., Lange, R., et al. (2017). Region and cell-type resolved quantitative proteomic map of the human heart. Nat Commun *8*, 1469. https://doi.org/10.1038/s41467-017-01747-2.

Farah, E.N., Hu, R.K., Kern, C., Zhang, Q., Lu, T.-Y., Ma, Q., Tran, S., Zhang, B., Carlin, D., Monell, A., et al. (2024). Spatially organized cellular communities form the developing human heart. Nature https://doi.org/10.1038/s41586-024-07171-z.

Frankish, A., Diekhans, M., Ferreira, A.-M., Johnson, R., Jungreis, I., Loveland, J., Mudge, J.M., Sisu, C., Wright, J., Armstrong, J., et al. (2019). GENCODE reference annotation for the human and mouse genomes. Nucleic Acids Research *47*, D766–D773. https://doi.org/10.1093/nar/gky955.

Garrido-Martín, D., Palumbo, E., Guigó, R., and Breschi, A. (2018). ggsashimi: Sashimi plot revised for browser- and annotation-independent splicing visualization. PLoS Comput Biol *14*, e1006360. https://doi.org/10.1371/journal.pcbi.1006360.

Hänzelmann, S., Castelo, R., and Guinney, J. (2013). GSVA: gene set variation analysis for microarray and RNA-Seq data. BMC Bioinformatics *14*. https://doi.org/10.1186/1471-2105-14-7.

Hao, Y., Hao, S., Andersen-Nissen, E., Mauck, W.M., Zheng, S., Butler, A., Lee, M.J., Wilk, A.J., Darby, C., Zager, M., et al. (2021). Integrated analysis of multimodal single-cell data. Cell *184*, 3573-3587.e29. https://doi.org/10.1016/j.cell.2021.04.048.

Igor Dolgalev (2025). msigdbr: MSigDB Gene Sets for Multiple Organisms in a Tidy Data Format.

Kolde R (2019). pheatmap: Pretty Heatmaps.

Krueger, F., James, F., Ewels, P., Afyounian, E., and Schuster-Boeckler, B. (2021). FelixKrueger/TrimGalore: v0.6.7 - DOI via Zenodo (Zenodo).

Li, H., Handsaker, B., Wysoker, A., Fennell, T., Ruan, J., Homer, N., Marth, G., Abecasis, G., Durbin, R., and 1000 Genome Project Data Processing Subgroup (2009). The Sequence Alignment/Map format and SAMtools. Bioinformatics *25*, 2078–2079. https://doi.org/10.1093/bioinformatics/btp352.

Louadi, Z., Elkjaer, M.L., Klug, M., Lio, C.T., Fenn, A., Illes, Z., Bongiovanni, D., Baumbach, J., Kacprowski, T., List, M., et al. (2021). Functional enrichment of alternative splicing events with NEASE reveals insights into tissue identity and diseases. Genome Biol *22*, 327. https://doi.org/10.1186/s13059-021-02538-1.

Love, M.I., Huber, W., and Anders, S. (2014). Moderated estimation of fold change and dispersion for RNA-seq data with DESeq2. Genome Biol *15*, 550. https://doi.org/10.1186/s13059-014-0550-8.

Maia, T.M., Staes, A., Plasman, K., Pauwels, J., Boucher, K., Argentini, A., Martens, L., Montoye, T., Gevaert, K., and Impens, F. (2020). Simple Peptide Quantification Approach for MS-Based Proteomics Quality Control. ACS Omega *5*, 6754–6762. https://doi.org/10.1021/acsomega.0c00080.

Melsted, P., Booeshaghi, A.S., Liu, L., Gao, F., Lu, L., Min, K.H., Da Veiga Beltrame, E., Hjörleifsson, K.E., Gehring, J., and Pachter, L. (2021). Modular, efficient and constant-memory single-cell RNA-seq preprocessing. Nat Biotechnol *39*, 813–818. https://doi.org/10.1038/s41587-021-00870-2.

Mercer, T.R., Neph, S., Dinger, M.E., Crawford, J., Smith, M.A., Shearwood, A.-M.J., Haugen, E., Bracken, C.P., Rackham, O., Stamatoyannopoulos, J.A., et al. (2011). The Human Mitochondrial Transcriptome. Cell *146*, 645–658. https://doi.org/10.1016/j.cell.2011.06.051.

Neuwirth E (2014). RColorBrewer: ColorBrewer Palettes.

Newman, A.M., Steen, C.B., Liu, C.L., Gentles, A.J., Chaudhuri, A.A., Scherer, F., Khodadoust, M.S., Esfahani, M.S., Luca, B.A., Steiner, D., et al. (2019). Determining cell type abundance and expression from bulk tissues with digital cytometry. Nat Biotechnol *37*, 773–782. https://doi.org/10.1038/s41587-019-0114-2.

participants in the 1st Human Cell Atlas Jamboree, Lun, A.T.L., Riesenfeld, S., Andrews, T., Dao, T.P., Gomes, T., and Marioni, J.C. (2019). EmptyDrops: distinguishing cells from empty droplets in droplet-based single-cell RNA sequencing data. Genome Biol *20*, 63. https://doi.org/10.1186/s13059-019-1662-y.

Patro, R., Duggal, G., Love, M.I., Irizarry, R.A., and Kingsford, C. (2017). Salmon provides fast and bias-aware quantification of transcript expression. Nat Methods *14*, 417–419. https://doi.org/10.1038/nmeth.4197.

R Core Team (2021). R: A language and environment for statistical computing (R Foundation for Statistical Computing, Vienna, Austria.).

Shen, S., Park, J.W., Lu, Z., Lin, L., Henry, M.D., Wu, Y.N., Zhou, Q., and Xing, Y. (2014). rMATS: Robust and flexible detection of differential alternative splicing from replicate RNA-Seq data. Proc. Natl. Acad. Sci. U.S.A. *111*. https://doi.org/10.1073/pnas.1419161111.

Soneson, C., Love, M.I., and Robinson, M.D. (2016). Differential analyses for RNA-seq: transcript-level estimates improve gene-level inferences. F1000Res *4*, 1521. https://doi.org/10.12688/f1000research.7563.2.

Suzuki, R., and Shimodaira, H. (2006). Pvclust: an R package for assessing the uncertainty in hierarchical clustering. Bioinformatics *22*, 1540–1542. https://doi.org/10.1093/bioinformatics/btl117.

Tapial, J., Ha, K.C.H., Sterne-Weiler, T., Gohr, A., Braunschweig, U., Hermoso-Pulido, A., Quesnel-Vallières, M., Permanyer, J., Sodaei, R., Marquez, Y., et al. (2017). An atlas of alternative splicing profiles and functional associations reveals new regulatory programs and genes that simultaneously express multiple major isoforms. Genome Res. *27*, 1759–1768. https://doi.org/10.1101/gr.220962.117.

Vaquero-Garcia, J., Aicher, J.K., Jewell, S., Gazzara, M.R., Radens, C.M., Jha, A., Norton, S.S., Lahens, N.F., Grant, G.R., and Barash, Y. (2023). RNA splicing analysis using heterogeneous and large RNA-seq datasets. Nat Commun *14*, 1230. https://doi.org/10.1038/s41467-023-36585-y.

Wei, Taiyun and Simko, Viktor R package “corrplot”: Visualization of a Correlation Matrix.

Wickham, H. (2016). ggplot2 (Cham: Springer International Publishing).

Wu, T., Hu, E., Xu, S., Chen, M., Guo, P., Dai, Z., Feng, T., Zhou, L., Tang, W., Zhan, L., et al. (2021). clusterProfiler 4.0: A universal enrichment tool for interpreting omics data. The Innovation *2*, 100141. https://doi.org/10.1016/j.xinn.2021.100141.
